# Connecting research and community: a methodological framework for investigating CMV transmission in childcare settings

**DOI:** 10.3389/fped.2025.1657706

**Published:** 2025-10-23

**Authors:** Karen Del’Olio, Annie Geiger, Judith Terry, Cindi Callaghan, Lauren Howe, Cheryl Hamel, Delaney Platia, Alyssa Blake, Lisa Tran, Anne Davenport, Elizabeth Orvek, Stephen Lammi, Bruce Barton, Timothy Kowalik, John Holik, Anne Mirza, Olesea Cojohari, Kelsey Woods, Susan Druker, Syed Saad Naeem, John D. Diaz-Decaro, Madeleine Hayden, Iliana Leony Lasso, Juli Gulpinar, Sandeep Basnet, Lori Panther, Andrew Natenshon, Thejas Suvarna, Emma Harman, Kelley Bridges, Summer Schrader, Laura Gibson

**Affiliations:** ^1^ Infectious Disease and Immunology, Departments of Medicine and of Pediatrics, UMass Chan Medical School, Worcester, MA, United States; ^2^ Academic Services Group, University of Massachusetts Chan Medical School, Worcester, MA, United States; ^3^ empHowered PR, LLC, Leominster, MA, United States; ^4^ Department of Population and Quantitative Health Sciences, UMass Chan Medical School, Worcester, MA, United States; ^5^ Department of Microbiology, UMass Chan Medical School, Worcester, MA, United States; ^6^ ModernaTX, Inc., Cambridge, MA, United States; ^7^ CareEvolution, Ann Arbor, MI, United States

**Keywords:** CMV, early education and care centers, CMV transmission, community engagement, diversity, equity and inclusion

## Abstract

The CMV Transmission and Immune Tracking (TransmIT) Study was developed to address critical gaps in understanding of cytomegalovirus (CMV) transmission dynamics in early education and care (EEC) settings. This two-stage, community-engaged study design integrates EEC center partnerships, digital study platforms, and data pipeline infrastructures to enable longitudinal virologic and immunologic surveillance in this high-exposure environment. Stage I focused on establishing foundational components of the study, including a geographically diverse EEC center network, culturally tailored recruitment strategies, a community advisory board, protocols for participant enrollment and saliva sample collection, and optimized laboratory assays to measure viral shedding in saliva. The study approach honed during Stage I is intended to support future longitudinal investigations into viral shedding patterns, immune responses, and co-infections among children and staff in EEC centers. This manuscript presents a methodological framework for conducting community-centered scalable research in early childhood settings with relevance for CMV and other infectious diseases of public health importance.

## 1 Introduction

Childcare centers provide essential services that support family and work life. However, any regular gathering of young children presents an opportunity for infectious diseases like conjunctivitis, respiratory infections, or gastroenteritis to circulate among children and their caretakers. A common pathogen in these settings is cytomegalovirus (CMV), a ubiquitous β-herpesvirus that infects people of all ages, especially children (1–3). CMV spreads via body fluids containing infectious virions, such as saliva and urine (4). Among children <3 years of age in childcare centers, the prevalence of CMV shedding in saliva or urine ranges from 10% to 70% (1). Moreover, high viral loads of CMV can continue to be shed well into early childhood up to 5 years of age (5), by which point about one-third of children in the United States have been infected (6). As a result, children attending large group programs are a major source of CMV transmission. Given their predictable exposure to saliva and urine of these children, caretakers at home or in childcare centers are at high risk for CMV exposure (7–18). If they are pregnant, CMV can disseminate to the fetus and lead to pregnancy loss or congenital CMV infection (cCMV) of live-born infants (8, 13, 16, 19–25).

CMV is not only the most common congenital infection worldwide, with a birth prevalence of 0.6%–0.7% (approximately 1 in 200 infants) in developed countries and 1%–5% in developing countries (26, 27), but also the leading infectious cause of birth defects and non-genetic cause of hearing loss (28, 29). An estimated 10%–15% of infants with CMV infection have clinically apparent symptoms at birth, such as microcephaly, hepatosplenomegaly, or cytopenias (30). Of the remainder born without visible symptoms, about 10%–15% will develop complications later in childhood, most commonly hearing loss but also neurodevelopmental delays (26, 27, 31–33). Nearly 20% of all infants with cCMV infection develop permanent long term effects (34). Despite the significant familial and societal burden of disease, awareness of cCMV is low (26, 29, 33, 35–39). Global surveys with data up to 2020 suggest that <7% of adults and <40% of pregnant women have heard of CMV (40–42). Furthermore, CMV transmission patterns and mechanisms of viral control in childcare settings remain poorly understood, limiting the development of educational materials and interventions to reduce cCMV prevalence (43).

The CMV Transmission and Immune Tracking (TransmIT) Study was developed to address gaps in understanding of CMV transmission in early education and care settings by establishing a research network through structured community engagement (44–49), integration of digital study platforms, and standardized laboratory methodologies (50–53). Our approach provides a scalable model for studying virologic and immunologic factors driving CMV shedding and transmission. This report details the methodology used in the early stages of the CMV TransmIT Study, with the potential to inform policy, clinical practice, and public health interventions for CMV and other infectious diseases.

## 2 Methods

### 2.1 Overall study design

In the absence of recent or local community-based studies in childcare centers, the CMV Transmission and Immune Tracking (TransmIT) Study was structured to progress in two stages. The initial stage was designed to establish key operational resources to support both long-term sustainability and future expansion to more complex studies and additional pathogens in this high-transmission setting.

Stage I focused on assembling a foundational infrastructure and building community relationships. Objectives included refining center and participant recruitment strategies, developing study materials, securing institutional review board (IRB) approvals, establishing data storage and flow processes, validating sample collection and experimental protocols, and addressing unique challenges in childcare settings for example, identifying appropriate, low-disruption areas within centers for sample collection or navigating variable pickup/drop-off times that complicated scheduling with families. By devising a relatively simple study protocol, Stage I not only allowed for iterative trials and refinement of critical workflows, but also enabled center staff and families to become familiar with the study team and the research process.

Stage II will expand on these activities through a longitudinal study that tracks CMV transmission dynamics and co-circulation with other pathogens. Initial aims are to characterize intra- and inter-host CMV populations, assess immune responses, and identify parameters associated with reduced CMV shedding over time. A platform study design will facilitate inclusion of other pathogens and co-infections commonly encountered in childcare settings, such as respiratory viruses.

**Figure 1:**
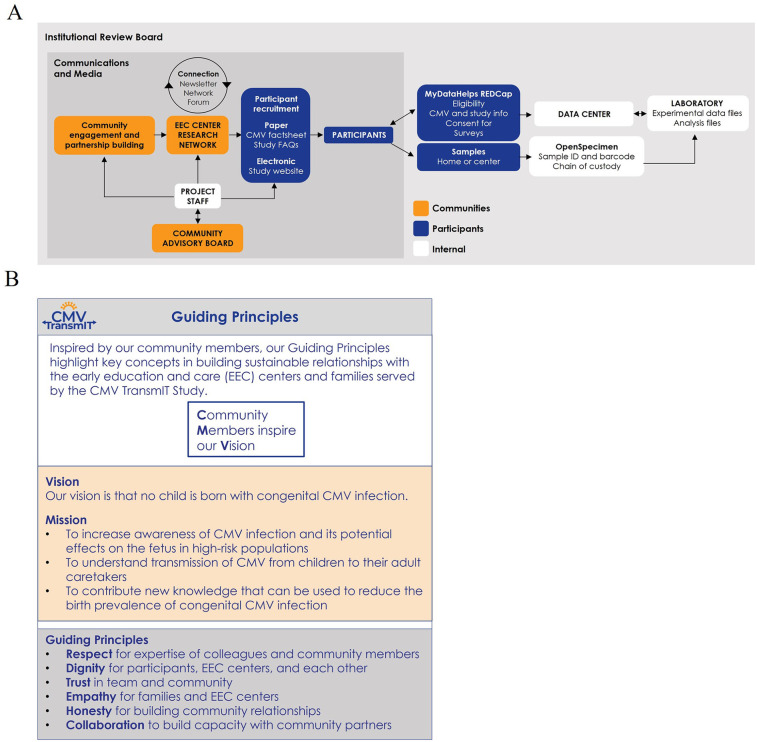
Components and guiding principles of stage I of the CMV transmIT study. **(A)** Conceptual flow and relationships between key components in the study. **(B)** The principles guiding the activities of the study. CMV, cytomegalovirus; EEC, early education and care; FAQs, frequently asked questions; ID, identifier; TransmIT, transmission and immune tracking.

This two-stage approach ensures that operational and methodological challenges and inefficiencies are optimized in a controlled manner during Stage I before launching more complex studies in Stage II. This method provides a replicable and scalable framework for investigating diverse infectious agents in group childcare environments and makes the work broadly applicable to public health research. The components and guiding principles of Stage I are summarized in Figure 1.

### 2.2 Community engagement and advisory structures

#### 2.2.1 Community advisory board

A Community Advisory Board was established to ensure that study activities aligned with community values and priorities. In consultation with the UMass Chan Medical School Community Engagement and Collaboration Core, a three-phase board member recruitment plan was implemented (Supplementary Appendixes 1A–C). The Board's objectives were to (1) advise on research design and implementation to enhance trust and acceptance, (2) ensure that protocols and materials respect community norms and traditions, (3) facilitate engagement and communication between the study team and community members, and (4) help maintain and expand community networks. The Board input also guided modifications to educational content, risk communication, and cultural considerations. These and other roles of the Board were detailed in its mission and charter (Supplementary Appendix 1D).

#### 2.2.2 Diversity, equity, and inclusion

The CMV TransmIT Study team viewed diversity, equity, and inclusion as fundamental elements of community engagement efforts. Given that culture, language, and socioeconomic context varied widely among early education and care (EEC) centers, education and recruitment materials were developed with consideration for health literacy, linguistic accessibility, and cultural values. For example, to support participation among Spanish-speaking families, selected study materials, including social media content, educational materials, and enrollment instructions were translated into Spanish. Board and local stakeholders were engaged to ensure that study processes respected community traditions and anticipated potential concerns. For example, feedback from center directors indicated that the preferred professional terminology in Massachusetts is “early education and care” rather than “daycare” or “childcare,” reflecting both the educational and caregiving missions of these institutions. By prioritizing equitable participation and fostering an atmosphere of mutual respect, we aimed to build a more inclusive research environment—one that could ultimately strengthen trust, broaden engagement, and maximize the relevance and applicability of study findings for all communities.

### 2.3 Early education and care (EEC) center network

At the onset of planning in 2018, a comprehensive search of the Massachusetts Department of Early Education and Care (MA DEEC) online directory identified 247 licensed large-group centers serving infants, toddlers, and preschoolers in the Worcester (*n* = 107) and Cambridge (*n* = 140) areas (52). From this pool, the study aimed to recruit approximately 20 public or private, non–home-based centers (about 10 per region). Early data collection included center characteristics such as maximum classroom size by age group (7 children for infants aged <15 months, 9 for toddlers aged 15–33 months, and 10 for preschoolers aged 33 months–pre-kindergarten (54) and infection control protocols. To strengthen center recruitment, community engagement experts with experience in education-based research were consulted to provide best practices and training for the study team.

**Figure 2:**
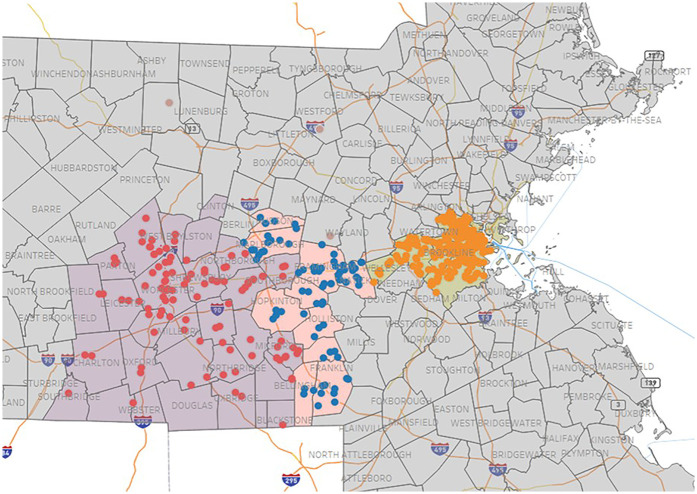
Regions and centers in Massachusetts included for recruitment for the CMV transmIT study. EEC centers in the Worcester (red), MetroWest (blue), and Cambridge/Boston (orange) regions were approached during recruitment to participate in the study. CMV, cytomegalovirus; EEC, early education and care; TransmIT, transmission and immune tracking.

**Figure 3:**
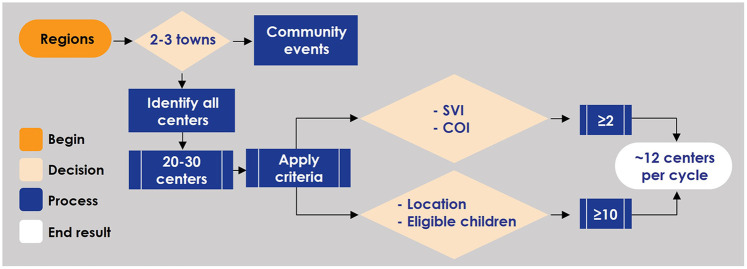
Center outreach strategy. EEC centers were identified for outreach based on the travel feasibility for field teams and the number of eligible children. Least resourced areas were intentionally identified and engaged based on SVI and COI data. COI, child opportunity index; EEC, early education and care; SVI, social vulnerability index.

The primary objective was to develop a sustainable EEC center research network emphasizing collaborative partnerships and minimizing operational burden on participating sites. Initial recruitment efforts were guided mainly by convenience factors, such as existing professional contacts, proximity to the study institution, and access to organizational networks linking multiple centers. An outreach database was developed in Research Electronic Data Capture (REDCap) (55, 56), integrating data from the Massachusetts Department of Early Education and Care (MA DEEC) directory and tracking real-time progress in center recruitment. As network expansion progressed, it became clear that relying exclusively on convenience-based strategies was insufficient to achieve a diverse and representative sample of centers and families. To address this, the sampling frame was expanded by grouping eastern Massachusetts into three broad regions—Worcester, MetroWest, and Cambridge/Boston (Figure 2). Centers were then identified for outreach based on travel feasibility for field teams and the number of children eligible for study enrollment. To avoid a bias toward better-resourced areas, the Collaboration Opportunities in Research Engagement (CORE) approach was developed to intentionally engage communities less likely to have access to, familiarity with, or participation in research. Despite some overlap in the underlying datasets, a pilot approach using Social Vulnerability Index (SVI) (57) and Child Opportunity Index (COI) data was used to identify and prioritize additional centers during outreach cycles. In the revised model (Figure 3), each outreach cycle involved contacting approximately 12–14 centers selected based on travel feasibility, enrollment potential, and, for at least 20% of centers, suboptimal SVI and COI metrics despite not meeting other eligibility criteria. This strategy also included collaboration with trusted community voices, participation in local cultural events and public forums, the delivery of accessible CMV education, and additional activities to cultivate authenticity, foster trust, and encourage meaningful participation across a wide range of communities in the CMV TransmIT Study.

### 2.4 Data management and infrastructure

Given trends toward real-time data collection (58) and to minimize barriers for participants such as paper documents or travel, digital study platforms were developed. MyDataHelps (MDH), created by CareEvolution, provided a website format to streamline the user experience of enrolling children and facilitate a low-friction onboarding mechanism for parents without the need to download an application or create an account (Supplementary Appendix 2). In addition to public-facing materials, a prominent “call-to-action” was included on the website home page to encourage visitors to connect with the study. Further exploration let parents view CMV and study information, determine eligibility, provide consent, validate relevant information, follow step-by-step instructions, and access on-site visit and sample collection schedules for their specific center. Home addresses were validated through the platform and identity of the child via parents or center staff. Links to surveys and other information were provided via email and/or mobile phone text message. Similarly, REDCap (55, 56) provided a straightforward platform for center staff to enroll and perform study activities. Contact information was displayed prominently on these platforms and other study materials to encourage communication with study staff. Overall, content required not only accessible language but also formatting, graphic design, and cohesiveness for an inviting experience.

**Figure 4:**
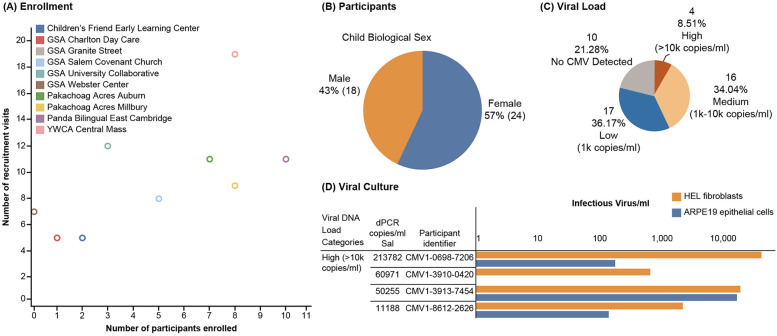
Study dashboard. Certain metrics are shown as examples for the **(A)** enrollment, **(B)** participants, **(C)** viral load, and **(D)** viral culture facets of the study dashboard. CMV, cytomegalovirus; dPCR, digital polymerase chain reaction; GSA, Guild of St Agnes; YWCA, Young Women's Christian Association.

Without an existing study infrastructure, conducting a needs assessment was essential to formulating the data management plan. The assessment considered security, access, format, storage, quality control, and data pipeline needs from all sources-to-storage. Given the relatively low sensitivity of data extracted from sources and moved through the system (i.e., very limited protected health information and personal identifiable information), data security was less of a concern, so the initial design focused on efficiency and scalability. The strategy included flexible evolution across both stages of the project, starting with data collected at one time point and preparing for subsequent longitudinal data collection at multiple time points. Successfully integrating data from multiple sources (e.g., MDH for children and REDCap for center staff, sample chain-of-custody and management platforms, and laboratory experiments) required careful planning to ensure consistent exports and accurate data linkage. The data management plan also included strict quality control measures to ensure data accuracy through pipelines and into analytical activities. The final solution was not only effective and flexible enough for evolving data collection, but also reliable and efficient enough for real-time analysis. A dashboard was created for easily viewing key study components (Figure 4).

### 2.5 Communications and media strategy

**Figure 5:**
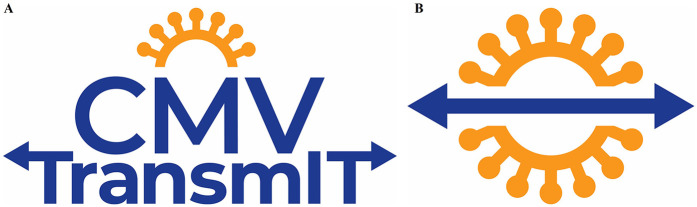
The CMV transmIT study logo **(A)** and graphic **(B)** the CMV transmIT study logo and graphic were among the branding features designed to promote recognition of the study and for use on electronic and printed materials. CMV, cytomegalovirus; TransmIT, transmission and immune tracking.

With the objective to increase CMV awareness and intention to disseminate study findings, we created multiple public access points. Communication and media strategies were developed in collaboration with the parent institution, funding sponsor, and public relations experts to align with best practices for branding, media engagement, and social responsibility (Supplementary Appendix 3). When appropriate, output was circulated for feedback and vetted with relevant UMass Chan Medical School and sponsor stakeholders prior to release. A logo, graphic, and other branding features were created to promote recognition (Figure 5). The study website was created not only for participant enrollment but also for public education about CMV. Other mechanisms included local and wire press releases, podcasts, monthly blogs, media briefings and pitches, thought leadership articles, and social media posts distributed through various channels. Training was provided for study personnel to ensure clear and consistent messaging to the media and public. Future efforts are planned to evaluate public engagement with these communication tools as part of future study activities.

### 2.6 Ethics considerations and IRB approval

The CMV TransmIT Study protocol and materials were developed per U.S. Department of Health and Human Services regulations for human subjects research (45 CFR 46) (54) and approved by the UMass Chan Medical School Institutional Review Board (IRB). Investigators, IRB staff, and study team members collaborated on iterative development of the Investigator Study Plan and informed consent form. Participant-facing materials were prepared in plain language (59) with attention to health literacy (60) and U.S. Centers for Disease Controls and Prevention health equity guidelines (60). Input from a National Institutes of Health Department of Bioethics representative guided decisions on reporting CMV polymerase chain reaction (PCR) results to participants. Consistent with that guidance, parents received education about CMV but did not receive results of saliva CMV PCR. CMV education highlighted that viral shedding in saliva is common in young children and that a positive PCR test would not change their usual healthcare or ability to continue at their center. Parents were also advised to contact their pediatrician for questions about CMV and their child specifically.

### 2.7 EEC center orientation and ongoing engagement

**Figure 6:**
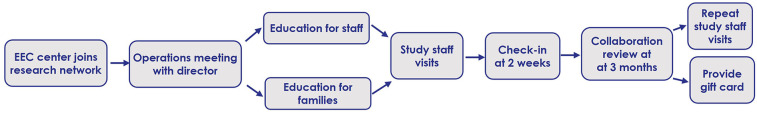
Workflow for orientation and engagement with EEC centers. Steps following center enrollment included director meetings, staff and family education, scheduled check-ins, and ongoing engagement activities to support study implementation. EEC, early education and care.

Our partnership with centers that joined the network began with an orientation meeting involving center directors and the study team (principal investigator, program manager, administrative coordinator, and research nurse; Figure 6). The conversation yielded a study operations plan customized to each center and a signed description of our collaboration to document roles and align expectations (Supplementary Appendix 4). The plan balanced IRB-approved activities with director preferences on study visit schedules, areas for meeting families and collecting samples, and other center-specific decisions. CMV education sessions for center staff and for families were provided in the format (in-person or virtual) or time (day or evening) requested by directors, thus aligning study activities with centers' familiar routines. Directors completed a survey on center demographics and practices (Supplementary Appendix 5). To provide benefit and build relationships with and among centers in the network, for center staff and families, we offered CMV education and access to the principal investigator for scientific questions, check-in and feedback sessions, annual gift cards, quarterly newsletters (Supplementary Appendix 6), group forums to provide study updates and share findings, and other appreciation efforts.

### 2.8 Participant recruitment and enrollment

Recruitment materials focused on CMV and study information and identified staff presence at centers (Supplementary Appendix 7). Study enrollment for Stage 1 was first limited to children <36 months of age attending EEC centers in the research network. Enrollment was later expanded to also include any staff member working at least 5 weeks per year, regardless of their role (56) with the primary goal of piloting staff participation procedures in anticipation for Stage II. Lack of exclusion criteria maintained inclusivity. The enrollment period was 4 weeks per participant to accommodate CMV education sessions, the informed consent process, demographic survey completion (Supplementary Appendix 8), and saliva sample collection. The Stage I enrollment goal was approximately five children per center at 20 centers (100 children total) and at least five staff members each at two centers (10 staff total), balancing representativeness and feasibility. Families were invited to share feedback and attend focus groups whether or not their child participated in the study (Supplementary Appendix 9).

### 2.9 Sample collection and laboratory procedures

A unique identification system for participants and samples (Supplementary Appendix 10) was designed to adapt to both stages of the project. The selection of saliva collection devices—Micro•SAL™ for children (later modified to include a volume indicator) and Super•SAL2™ for adults—was based on predefined criteria (Supplementary Appendix 11). One saliva sample per participant was collected either on-site by study staff or through a pilot home collection option. The pilot evaluated feasibility, logistics, and acceptability of home sample collection, planned enrollment of at least 15 children and 15 EEC center staff (no more than 60 total) and included participant feedback on home sampling safety and experience (Supplementary Appendixes 12–S13). Results from the pilot will inform Stage II sample collection procedures.

All saliva samples underwent standardized workflows for receipt, processing, and storage. Digital PCR (dPCR) assays were optimized to measure the absolute number of CMV DNA molecules per sample, which defined CMV shedding prevalence at each center. A CMV DNA standard was used to determine the limit of detection (LOD) for the dPCR assay. Results were expressed in copies/ml rather than IU/ml, as the assay was optimized for research use only and not intended as a clinical diagnostic. A viral infectivity assay was developed to quantify the level of infectious CMV in each sample and test viral tropism for fibroblast and/or epithelial cells. Known viral stocks were used to determine the LOD of this assay.

## 3 Anticipated results

The staged approach of CMV TransmIT is expected to yield a range of informative outcomes. Stage I will establish a robust research network and demonstrate the feasibility of recruiting participants and collecting saliva samples in the EEC setting. Evolving center outreach and community engagement activities, guided by established principles, the CORE center outreach strategy, and input from the Community Advisory Board, are intended to provide CMV education to people capable of becoming pregnant, increase access to research in general, and strengthen trust within EEC centers and their surrounding communities. Such fundamental efforts increase the probability, quality, and sustainability of partnerships with individual centers and their consortium that can lead to mutually beneficial interactions and increasing enrollment and retention rates.

The integration of MDH and REDCap platforms is anticipated to streamline participant onboarding, facilitate reliable data linkage, and enable real-time monitoring of enrollment, sample collection, and key laboratory data. Measurements of CMV in saliva obtained through digital PCR and viral infectivity assays are expected to demonstrate that children under 3 years of age in large-group early education programs frequently shed CMV in saliva and may serve as sources of infection for individuals who are pregnant.

In the longer term, the foundational structure and data from Stage I are expected to inform the design and implementation of more complex, longitudinal studies in Stage II. Learnings from Stage I will enhance operational efficiency and support the development of new collaborations with centers and other partners. Expanding study staff presence at centers and within their local communities is anticipated to improve familiarity and possibly reduce discomfort with research participation. Additionally, targeted participant enrollment strategies, refined sampling procedures, and validated laboratory methods will strengthen future study designs and enable robust hypothesis testing. Stage II will also expand investigation to include other pathogens relevant to early education and care settings, ultimately guiding a more comprehensive understanding of viral transmission patterns and immune responses.

## 4 Discussion

The CMV TransmIT Study was designed to address critical gaps in understanding of CMV transmission dynamics in EEC settings. Multiple studies have suggested that children under 3 years of age are a major source of infectious virus for pregnant caretakers and may therefore be an effective target population for a CMV vaccine (61, 62). The central premise of the study is that reducing CMV spread from young children to people who are pregnant will ultimately decrease the birth prevalence of cCMV. The path to achieving this goal is complicated by limited public awareness, incomplete knowledge of CMV natural history in childcare contexts, and the operational complexities of community-based research (63). To begin navigating these issues, a two-stage study was implemented that first focused on community engagement, key infrastructure components, and laboratory methodologies. From the outset, community engagement was integral to building local capacity and ensuring that the research reflected community values and priorities (57). Stakeholders were consulted to understand the current EEC landscape, engaged a Community Advisory Board with varied expertise, and incorporated principles of diversity, equity, and inclusion into participant-facing materials (58). Center-specific onboarding processes were tailored to the unique context and prior research experience of each community, promoting shared decision-making and fostering early trust. Engagement with families, staff, and local communities contributed to the establishment of an expanding research network of EEC centers. Concurrently, the development of internal staffing, technological systems, and laboratory workflows necessitated the creation of a multidimensional research infrastructure.

The study began with a relatively simple protocol—a brief survey and a single saliva sample—to introduce the research process in a non-intrusive manner and to demonstrate the feasibility of conducting research in EEC centers. Collecting one sample per participant provided sufficient volume while maintaining a manageable pace for developing essential biosafety, experimental, and data workflows.

The CMV TransmIT Study model offers many key advantages. While seemingly less efficient, the staged approach provided time for essential research components—community partnership and infrastructure development—to evolve gradually and sustainably. Building meaningful, trust-based relationships with centers required substantial time and nuanced understanding of their cultural, geographic, and familial contexts. The community-driven engagement and pace offered contextual insights that will enhance the relevance and impact of findings and future studies. Tailoring operations plans for each center allowed their leaders to guide implementation, thus reducing apprehension and ensuring that study activities accommodated their norms and routines. The scalable digital infrastructure supported efficient data collection, management, and quality assurance. Overall, we envision the study model will serve as an adaptable platform for investigating other infectious diseases and research questions in EEC settings.

As intended for this early stage, several insights emerged that required ongoing refinement of our approach or that arose from the nature of the EEC center setting. Real-time process improvement cycles (59) guided by study team insights and by feedback from participating centers or the Board were implemented frequently. Most significantly, a detailed understanding of certain communities prompted adoption of the CORE outreach strategy to more intentionally include sites with limited access to or familiarity with research, thereby enhancing inclusivity and relevance of the study for families utilizing EEC services. Similarly, we learned that integration of study staff with routine center activities during site visits—not only for participant recruitment but also for offering general support as needed—was instrumental in building trust and engagement. We discovered that familiarity with CMV among center staff and families was generally low, potentially reducing understanding and perceived relevance of the project. The resource intensive study approach, geographic constraints and in-person sample collection may have restricted center, family, or staff participation. The initial simplicity of the study protocol, while valuable for feasibility assessments, may only partially capture the complexities of CMV transmission patterns and immune responses. Outside of the study, many EEC centers faced operational demands on their attention and resources preventing or limiting their engagement in research. Given these intrinsic challenges, the primary obstacle to future studies will probably be low center or participant recruitment and retention. Some tactics to counteract this issue include increasing community engagement through collaborations with other researchers, existing resources, and local community leaders.

With Stage I completed, a versatile research infrastructure has been established, and the EEC center research network continues to expand. These efforts have yielded valuable insights into effective study implementation and have strengthened the network's readiness for more complex, longitudinal investigations. Lessons learned—from community engagement strategies to data and laboratory workflows—will guide future CMV transmission and immunity research in Stage II. Beyond CMV, the collaborative relationships formed with EEC centers provide a platform for investigating other infectious diseases of public health relevance. Leveraging this network, future studies may deploy new protocols, evaluate interventions, and generate evidence to inform policies and clinical practices aimed at improving child and community health. Importantly, Stage I underscored that research in community settings advances at the speed of trust (60) and depends on respect for local priorities and an adaptable mindset. These principles will continue to guide the evolution of the CMV TransmIT Study and inform its broader application to research on CMV and other pathogens.

## Data Availability

The data that support the findings of this study are not publicly available due to participant privacy and institutional restrictions. De-identified or summary data may be made available from the corresponding author upon reasonable request and with appropriate institutional approvals once the study is complete.

## References

[1] Collins JP Shane A. Infections associated with group childcare. In: Principles and Practice of Pediatric Infectious Diseases. 5th ed. Philadelphia, PA: Elsevier (2018) 25-32:e3.

[2] Gupta M Shorman M. Cytomegalovirus. In: StatPearls. Treasure Island, FL (2024).

[3] Centers for Disease Control and Prevention. Clinical Overview. Atlanta, GA: U.S. Department of Health and Human Services, Centers for Disease Control and Prevention (2022). Available online at: https://www.cdc.gov/cmv/clinical/overview.html (Accessed May 8, 2024).

[4] Centers for Disease Control and Prevention. About Cytomegalovirus (CMV). Atlanta, GA: U.S. Department of Health and Human Services, Centers for Disease Control and Prevention (2020). Available online at: https://www.cdc.gov/cmv/overview.html (August 18, 2023).

[5] Cannon MJ Hyde TB Schmid DS. Review of cytomegalovirus shedding in bodily fluids and relevance to congenital cytomegalovirus infection. Rev Med Virol. (2011) 21(4):240–55. 10.1002/rmv.695 21674676 PMC4494736

[6] Centers for Disease Control and Prevention. Cytomegalovirus (CMV) and Congenital CMV Infection—Clinical Overview. Atlanta, GA: U.S. Department of Health and Human Services, Centers for Disease Control and Prevention (2020). Available online at: https://www.cdc.gov/cmv/clinical/overview.html (Accessed October 26, 2023).

[7] Pass RF Hutto C Ricks R Cloud GA. Increased rate of cytomegalovirus infection among parents of children attending day-care centers. N Engl J Med. (1986) 314:1414–8. 10.1056/NEJM198605293142204 3010113

[8] Alain S Garnier-Geoffroy F Labrunie A Montané A Marin B Gatet M Cytomegalovirus (CMV) shedding in French day-care centers: a nationwide study of epidemiology, risk factors, centers’ practices, and parents’ awareness of CMV. J Pediatr Infect Dis Soc. (2020) 9(6):686–94. 10.1093/jpids/piz097 32068854

[9] Pass RF Little EA Stagno S Britt WJ Alford CA. Young children as a probable source of maternal and congenital cytomegalovirus infection. N Engl J Med. (1987) 316(22):1366–70. 10.1056/NEJM198705283162203 3033505

[10] Stagno S Cloud G Pass RF Britt WJ Alford CA. Factors associated with primary cytomegalovirus infection during pregnancy. J Med Virol. (1984) 13(4):347–53. 10.1002/jmv.1890130405 6330288

[11] US Department of Labour Occupational Safety and Health Administration. Cytomegalovirus. Washington, DC: U.S. Department of Labor, Occupational Safety and Health Administration. Available online at: https://www.osha.gov/cytomegalovirus (Accessed April 18, 2024).

[12] US Department of Labour Occupational Safety and Health Administration. Cytomegalovirus Control and Prevention. Washington, DC: U.S. Department of Labour, Occupational Safety and Health Administration. Available online at: https://www.osha.gov/cytomegalovirus/control-prevention (Accessed April 18, 2024).

[13] Romero Starke K Kofahl M Freiberg A Schubert M Groß ML Schmauder S The risk of cytomegalovirus infection in daycare workers: a systematic review and meta-analysis. Int Arch Occup Environ Health. (2020) 93(1):11–28. 10.1007/s00420-019-01464-x 31359142

[14] Adler SP. Molecular epidemiology of cytomegalovirus: evidence for viral transmission to parents from children infected at a day care center. Pediatr Infect Dis J. (1986) 5(3):315–8. 10.1097/00006454-198605000-00008 3014453

[15] Adler SP. Cytomegalovirus and child day care. Evidence for an increased infection rate among day-care workers. N Engl J Med. (1989) 321(19):1290–6. 10.1056/NEJM198911093211903 2552316

[16] Fowler KB Pass RF. Risk factors for congenital cytomegalovirus infection in the offspring of young women: exposure to young children and recent onset of sexual activity. Pediatrics. (2006) 118(2):e286–92. 10.1542/peds.2005-1142 16847076

[17] Balegamire SJ McClymont E Croteau A Dodin P Gantt S Besharati AA Prevalence, incidence, and risk factors associated with cytomegalovirus infection in healthcare and childcare worker: a systematic review and meta-analysis. Syst Rev. (2022) 11(1):131. 10.1186/s13643-022-02004-4 35754052 PMC9235282

[18] Zheng QY Huynh KT van Zuylen WJ Craig ME Rawlinson WD. Cytomegalovirus infection in day care centres: a systematic review and meta-analysis of prevalence of infection in children. Rev Med Virol. (2019) 29(1):e2011. 10.1002/rmv.2011 30306730

[19] Strangert K Carlström G Jeansson S Nord CE. Infections in preschool children in group day care. Acta Paediatr Scand. (1976) 65(4):455–63. 10.1111/j.1651-2227.1976.tb04914.x 779398

[20] Adler SP. Cytomegalovirus and child day care: risk factors for maternal infection. Pediatr Infect Dis J. (1991) 10(8):590–4. 10.1097/00006454-199108000-00008 1653939

[21] Balegamire SJ Renaud C Masse B Zinszer K Gantt S Giguere Y Frequency, timing and risk factors for primary maternal cytomegalovirus infection during pregnancy in Quebec. PLoS One. (2021) 16(6):e0252309. 10.1371/journal.pone.0252309 34170911 PMC8232530

[22] Williams EJ Embleton ND Clark JE Bythell M Platt MPW Berrington JE. Viral infections: contributions to late fetal death, stillbirth, and infant death. J Pediatr. (2013) 163(2):424–8. 10.1016/j.jpeds.2013.02.004 23507026

[23] Odendaal H Wright C Brink L Schubert P Geldenhuys E Groenewald C. Association of late second trimester miscarriages with placental histology and autopsy findings. Eur J Obstet Gynecol Reprod Biol. (2019) 243:32–5. 10.1016/j.ejogrb.2019.10.024 31670146 PMC6876705

[24] Syridou G Spanakis N Konstantinidou A Piperaki ET Kafetzis D Patsouris E Detection of cytomegalovirus, parvovirus B19 and herpes simplex viruses in cases of intrauterine fetal death: association with pathological findings. J Med Virol. (2008) 80(10):1776–82. 10.1002/jmv.21293 18712818

[25] Iwasenko JM Howard J Arbuckle S Graf N Hall B Craig ME Human cytomegalovirus infection is detected frequently in stillbirths and is associated with fetal thrombotic vasculopathy. J Infect Dis. (2011) 203(11):1526–33. 10.1093/infdis/jir121 21592980

[26] Akpan US Pillarisetty LS. Congenital cytomegalovirus infection, In: StatPearls. Treasure Island, FL (2024).

[27] Boucoiran I Yudin M Poliquin V Caddy S Gantt S Castillo E. Guideline no. 420: cytomegalovirus infection in pregnancy. J Obstet Gynaecol Can. (2021) 43(7):893–908. 10.1016/j.jogc.2021.05.015 34089905

[28] Salomè S Corrado F Mazzarelli L Maruotti G Capasso L Blazquez-Gamero D Congenital cytomegalovirus infection: the state of the art and future perspectives. Front Pediatr. (2023) 11:1276912. 10.3389/fped.2023.1276912 38034830 PMC10687293

[29] Ssentongo P Hehnly C Birungi P Roach MA Spady J Fronterre C Congenital cytomegalovirus infection burden and epidemiologic risk factors in countries with universal screening: a systematic review and meta-analysis. JAMA Netw Open. (2021) 4(8):e2120736. 10.1001/jamanetworkopen.2021.20736 34424308 PMC8383138

[30] Rawlinson WD Boppana SB Fowler KB Kimberlin DW Lazzarotto T Alain S Congenital cytomegalovirus infection in pregnancy and the neonate: consensus recommendations for prevention, diagnosis, and therapy. Lancet Infect Dis. (2017) 17(6):e177–88. 10.1016/S1473-3099(17)30143-3 28291720

[31] Boppana SB Ross SA Fowler KB. Congenital cytomegalovirus infection: clinical outcome. Clin Infect Dis. (2013) 57(suppl 4):S178–81. 10.1093/cid/cit629 24257422 PMC4471438

[32] Dollard SC Grosse SD Ross DS. New estimates of the prevalence of neurological and sensory sequelae and mortality associated with congenital cytomegalovirus infection. Rev Med Virol. (2007) 17(5):355–63. 10.1002/rmv.544 17542052

[33] Centers for Disease Control and Prevention. Babies Born with Congenital CMV. Atlanta, GA: U.S. Department of Health and Human Services, Centers for Disease Control and Prevention (2022). Available online at: https://www.cdc.gov/cmv/congenital-infection.html (Accessed February 4, 2024).

[34] Cannon MJ. Congenital cytomegalovirus (CMV) epidemiology and awareness. J Clin Virol. (2009) 46(Suppl 4):S6–10. 10.1016/j.jcv.2009.09.002 19800841

[35] Tastad KJ Schleiss MR Lammert SM Basta NE. Awareness of congenital cytomegalovirus and acceptance of maternal and newborn screening. PLoS One. (2019) 14(8):e0221725. 10.1371/journal.pone.0221725 31449545 PMC6709948

[36] Raines K Heitman KN Leung J Woodworth KR Tong VT Sugerman DE Congenital cytomegalovirus surveillance in the United States. Birth Defects Res. (2023) 115(1):11–20. 10.1002/bdr2.2098 36193579

[37] Gugliesi F Coscia A Griffante G Galitska G Pasquero S Albano C Where do we stand after decades of studying human cytomegalovirus? Microorganisms. (2020) 8(5):685. 10.3390/microorganisms8050685 32397070 PMC7284540

[38] Zappas MP Devereaux A Pesch MH. The psychosocial impact of congenital cytomegalovirus on caregivers and families: lived experiences and review of the literature. Int J Neonatal Screen. (2023) 9(2):30. 10.3390/ijns9020030 37367211 PMC10299480

[39] Benou S Dimitriou G Papaevangelou V Gkentzi D. Congenital cytomegalovirus infection: do pregnant women and healthcare providers know enough? A systematic review. J Matern Fetal Neonatal Med. (2022) 35(25):6566–75. 10.1080/14767058.2021.1918088 33944654

[40] Doutre SM Barrett TS Greenlee J KR W. Losing ground: awareness of congenital cytomegalovirus in the United States. J Early Hear Detect Interv. (2016) 1(2):39–48. 10.15142/T32G62

[41] Castillo K Hawkins-Villarreal A Valdés-Bango M Guirado L Scazzocchio E Porta O Congenital cytomegalovirus awareness and knowledge among health professionals and pregnant women: an action towards prevention. Fetal Diagn Ther. (2022) 49((5-6):265–72. 10.1159/000525528 35705068

[42] Bartnik P Bender A Kacperczyk-Bartnik J Ciebiera M Urban A Sienko A Awareness of pregnant patients about congenital cytomegalovirus infection-a semi-systematic review. J Clin Med. (2024) 13(9):2586. 10.3390/jcm13092586 38731115 PMC11084167

[43] Britt WJ. Human cytomegalovirus infection in women with preexisting immunity: sources of infection and mechanisms of infection in the presence of antiviral immunity. J Infect Dis. (2020) 221(Suppl 1):S1–8. 10.1093/infdis/jiz464 32134479 PMC7057782

[44] De Weger E Van Vooren N Luijkx KG Baan CA Drewes HW. Achieving successful community engagement: a rapid realist review. BMC Health Serv Res. (2018) 18(1):285. 10.1186/s12913-018-3090-1 29653537 PMC5899371

[45] Santillan DA Brandt DS Sinkey R Scheib S Peterson S LeDuke R Barriers and solutions to developing and maintaining research networks during a pandemic: an example from the iELEVATE perinatal network. J Clin Transl Sci. (2022) 6(1):e56. 10.1017/cts.2022.5 35720965 PMC9161042

[46] Wangen M Escoffery C Fernandez ME Friedman DB Hannon P Ko LK Twenty years of capacity building across the cancer prevention and control research network. Cancer Causes Control. (2023) 34(Suppl 1):45–56. 10.1007/s10552-023-01690-2 37067700 PMC10106885

[47] Riccardi MT Pettinicchio V Di Pumpo M Altamura G Nurchis MC Markovic R Community-based participatory research to engage disadvantaged communities: levels of engagement reached and how to increase it. A systematic review. Health Policy. (2023) 137:104905. 10.1016/j.healthpol.2023.104905 37716190

[48] Osborne J Paget J Giles-Vernick T Kutalek R Napier D Baliatsas C Community engagement and vulnerability in infectious diseases: a systematic review and qualitative analysis of the literature. Soc Sci Med. (2021) 284:114246. 10.1016/j.socscimed.2021.114246 34311391

[49] Cope EL McTigue KM Forrest CB Carton TW Fair AM Goytia C Stakeholder engagement infrastructure to support multicenter research networks: advances from the clinical research networks participating in PCORnet. Learn Health Syst. (2023) 7(1):e10313. 10.1002/lrh2.10313 36654809 PMC9835038

[50] Tan RK Wu D Day S Zhao Y Larson HJ Sylvia S Digital approaches to enhancing community engagement in clinical trials. NPJ Digit Med. (2022) 5(1):37. 10.1038/s41746-022-00581-1 35338241 PMC8956701

[51] Daniore P Nittas V von Wyl V. Enrollment and retention of participants in remote digital health studies: scoping review and framework proposal. J Med Internet Res. (2022) 24(9):e39910. 10.2196/39910 36083626 PMC9508669

[52] Sehrawat O Noseworthy PA Siontis KC Haddad TC Halamka JD Liu H. Data-driven and technology-enabled trial innovations toward decentralization of clinical trials: opportunities and considerations. Mayo Clin Proc. (2023) 98(9):1404–21. 10.1016/j.mayocp.2023.02.003 37661149

[53] Harmon DM Noseworthy PA Yao X. The digitization and decentralization of clinical trials. Mayo Clin Proc. (2023) 98(10):1568–78. 10.1016/j.mayocp.2022.10.001 36669937 PMC12056663

[54] Massachusetts Department of Early Education and Care. 606 CMR 7: Standards for the licensure or approval of family child care; small group and school age; and large group and school age child care programs (Mass. Register #1459). Boston, MA: Commonwealth of Massachusetts (2021).

[55] Harris PA Taylor R Thielke R Payne J Gonzalez N Conde JG. Research electronic data capture (REDCap)–a metadata-driven methodology and workflow process for providing translational research informatics support. J Biomed Inform. (2009) 42(2):377–81. 10.1016/j.jbi.2008.08.010 18929686 PMC2700030

[56] Harris PA Taylor R Minor BL Elliott V Fernandez M O’Neal L The REDCap consortium: building an international community of software platform partners. J Biomed Inform. (2019) 95:103208. 10.1016/j.jbi.2019.103208 31078660 PMC7254481

[57] Flanagan BE Hallisey EJ Adams E Lavery A. Measuring community vulnerability to natural and anthropogenic hazards: the centers for disease control and prevention’s social vulnerability index. J Environ Health. (2018) 80(10):34–6. PMID: 32327766 32327766 PMC7179070

[58] Goodson N Wicks P Morgan J Hashem L Callinan S Reites J. Opportunities and counterintuitive challenges for decentralized clinical trials to broaden participant inclusion. NPJ Digit Med. (2022) 5(1):58. 10.1038/s41746-022-00603-y 35513479 PMC9072305

[59] Clinical and Translational Science Awards Consortium Community Engagement Key Function Committee Task Force on the Principles of Community Engagement. Principles of Community Engagement. 2nd ed. Washington, DC: National Institutes of Health (2011). p. 45–53. NIH Publication No. 11-7782.

[60] Calanan RM Bonds ME Bedrosian SR Laird SK Satter D Penman-Aguilar A CDC’s guiding principles to promote an equity-centered approach to public health communication. Prev Chronic Dis. (2023) 20:E57. 10.5888/pcd20.230061 37410939 PMC10364834

[61] Lantos PM Gantt S Janko M Dionne F Permar SR Fowler K. A geographically weighted cost-effectiveness analysis of newborn cytomegalovirus screening. Open Forum Infect Dis. (2024) 11(6):ofae311. 10.1093/ofid/ofae311 38933739 PMC11200186

[62] Byrne C Coombs D Gantt S. Modestly protective cytomegalovirus vaccination of young children effectively prevents congenital infection at the population level. Vaccine. (2022) 40(35):5179–88. 10.1016/j.vaccine.2022.07.026 35907677

[63] Mussi-Pinhata MM Yamamoto AY. Natural history of congenital cytomegalovirus infection in highly seropositive populations. J Infect Dis. (2020) 221(Suppl 1):S15–22. 32134482 10.1093/infdis/jiz443 PMC7057789

