# Supplementary material for: Connecting research and community: a methodological framework for investigating CMV transmission in childcare settings
Source: Front Pediatr. 2025 Oct 23;13:1657706. doi: 10.3389/fped.2025.1657706 (PMC12589089; doi:10.3389/fped.2025.1657706)
Supplement: Supplementary file 1 [file Datasheet1.pdf]

1 Supplementary Material

Appendix 1

A. Phases of development of the Community Advisory Board for the CMV TransmIT Study.

Phase	Methods
Exploratory	• Literature was reviewed to understand the role of community advisory boards in clinical research, including the development of best practices and their relationship with research teams<br>• Key informant interviews were conducted with individuals in the EEC field. Informants completed an online survey and a 30-minute virtual interview with standardized questions about optimal messaging, expertise representation, and any potential concerns about joining the board
Invitational	• Based on feedback from the exploratory phase, categories of expertise were defined, such as parents of a child with cCMV, parents of a child attending a large group program, childcare center staff members, a community researcher, a professional in the hearing loss field, an occupational health professional, and others<br>• Emails describing the study and inviting recipients to join the board were then sent to individuals in each category of expertise
Organizational	• The first Board meeting convened after five members had accepted the invitation. Education about CMV and the study were provided.<br>• Board members completed a pre- and post-meeting survey to assess gaps in their knowledge and understanding of CMV and to gather feedback on proposed educational content for EEC center staff.<br>• Board members were also asked to complete an operations survey to gather their preferences for communication mechanisms, meeting schedule, document platform sharing, and other logistical details.<br>• A charter was developed including member roles, terms, benefits, decision-making processes, and meeting formats

cCMV, congenital CMV; CMV, cytomegalovirus; EEC, early education and care.

B. Results of interviews conducted with key informants during the exploratory phase.

Community Advisory Board Members		EEC Centers	
**Potential interest in joining the board (*N* = *6*)**	*n* (%)	**Potential interests in joining the study (*N* = *6*)**	*n* (%)
Passionate about CMV awareness	4 (67%)	Receipt of compensation, benefits, or incentives	3 (50%)
Receipt of compensation, benefits, or incentives	2 (33%)	Training and resources	1 (17%)
Knowledge of benefiting community	1 (17%)	Supporting interests of parents	1 (17%)
		Association with Moderna	1 (17%)
**Potential concerns about joining the board (*N* = *6*)**	*n* (%)	**Potential concerns about joining the study (*N* = *6*)**	*N (%)*
Lack of available time	5 (83%)	Fear surrounding CMV – centers want to avoid frightening employees and parents	2 (33%)
Meeting format (hybrid, virtual, or flexible)	3 (50%)	Additional tasks for employees to perform, or pandemic fatigue	3 (50%)
Lack of awareness about CMV	1 (17%)	Lack of awareness about CMV	2 (33%)
		Blood sample collection during Stage II may be upsetting to children and parents	2 (33%)

CMV, cytomegalovirus; EEC, early education and care.

C. Results of the board operations survey conducted during the organizational phase. The bold categories represent the most common responses to each question.

Question (*N* = *6*)	Responses	*n* (%)
**Single-answer questions**		
How would you like to receive updates from the CMV TransmIT Study team?	**Email**	**6 (100%)**
	Text	0 (0%)
	Newsletter	0 (0%)
	Social media	0 (0%)
	Other	0 (0%)
How often would you like to receive updates from the CMV TransmIT Study team?	**Monthly**	**3 (50%)**
	Quarterly	0 (0%)
	**As needed**	**3 (50%)**
	Other	0 (0%)
**Multiple-answer questions**		
What are the best days for you to attend quarterly board meetings?	Monday	3 (50%)
	**Tuesday**	**4 (66%)**
	Wednesday	3 (50%)
	**Thursday**	**4 (66%)**
	Friday	3 (50%)
Please select the best times of day for you to attend board meetings.	**Early morning**	**4 (66%)**
	Late morning	2 (33%)
	**Lunch time**	**4 (66%)**
	Early afternoon	2 (33%)
	Late afternoon	2 (33%)
	Evening	3 (50%)
What platform(s) would you like to use for working documents?	Share Point	0 (0%)
	Drop Box	2 (33%)
	**Google Docs**	**6 (100%)**
	Other	2 (33%)
Please use the space provided to share any additional resources, networks, or any other feedback.		1 (17%)

CMV, cytomegalovirus; TransmIT, transmission and immune tracking.

D. Mission statement and charter detailing the roles of the Community Advisory Board.

MISSION STATEMENT

The mission of the CMV Transmission and Immune Tracking (TransmIT) Study Community Advisory Board is to partner and provide expertise to the CMV TransmIT Study team to include early education and care centers representative and inclusive of the study's geographical communities.

CHARTER

The Community Advisory Board (Board) is a group of diverse individuals founded to represent the interests of the community and those potentially impacted by this study. The Charter of the Board will be to build capacity in the community and strengthen partnerships between community members and the research study team.

The Board will:

- Work with the research team to advise on recruitment and retention of early education and care (EEC) centers
- Bridge communications between research study team and community members
- Advise the research team to ensure dissemination of participant-facing materials are culturally inclusive
- Enhance and strengthen existing connections and build relationship of EEC centers in the of study research network
- Represent needs and interests of the community
- Ensure research practices are inclusive
- Engage the community and help spread awareness about the research
- Develop goals for work that assists the Board to achieve its mission

Appendix 2. Participant experience of the digital study platform. (**A**) Flyers for LARs were available at EEC centers, provided in children's backpacks, or by other means of communication for each center. The flyer contained a QR code that links to the CMV TransmIT Study website for enrolment information. (**B**) The digital study platform as experienced by the participants during the processes of enrolment, onboarding, eligibility assessment, consent, and selection of preferences for study procedures. (**C**) Flow of procedures from the completion of the consent form via MDH through to the receipt of a welcome email differed depending on whether the participant opted for sample collection at home or at the EEC center. Emails were sent weekly for any incomplete study activities.

CMV, cytomegalovirus; LAR, legally authorized representative; MDH, MyDataHelps; TransmIT, transmission and immune tracking; QR, quick response.

(A)

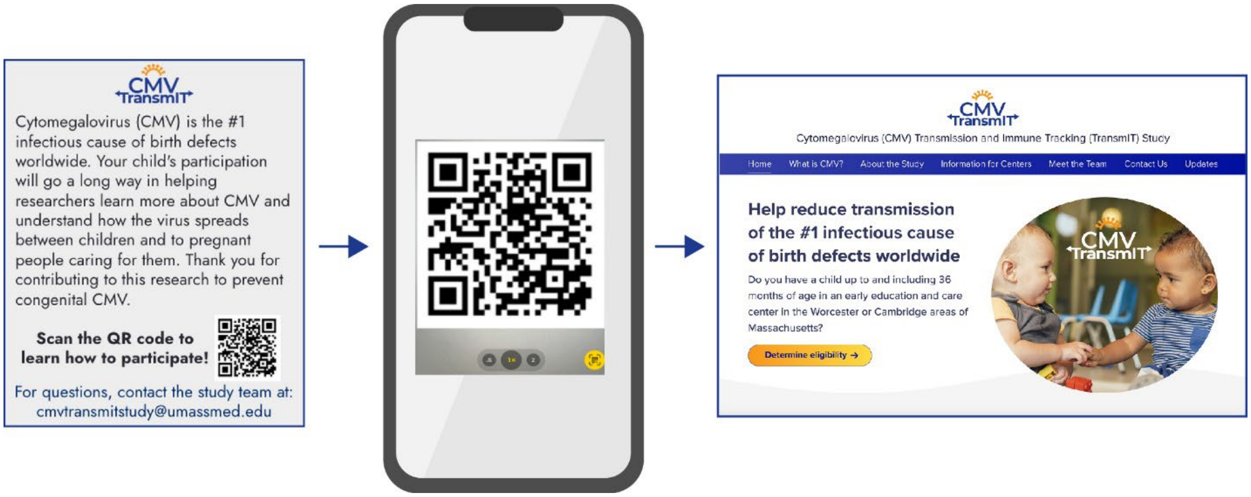

(B)

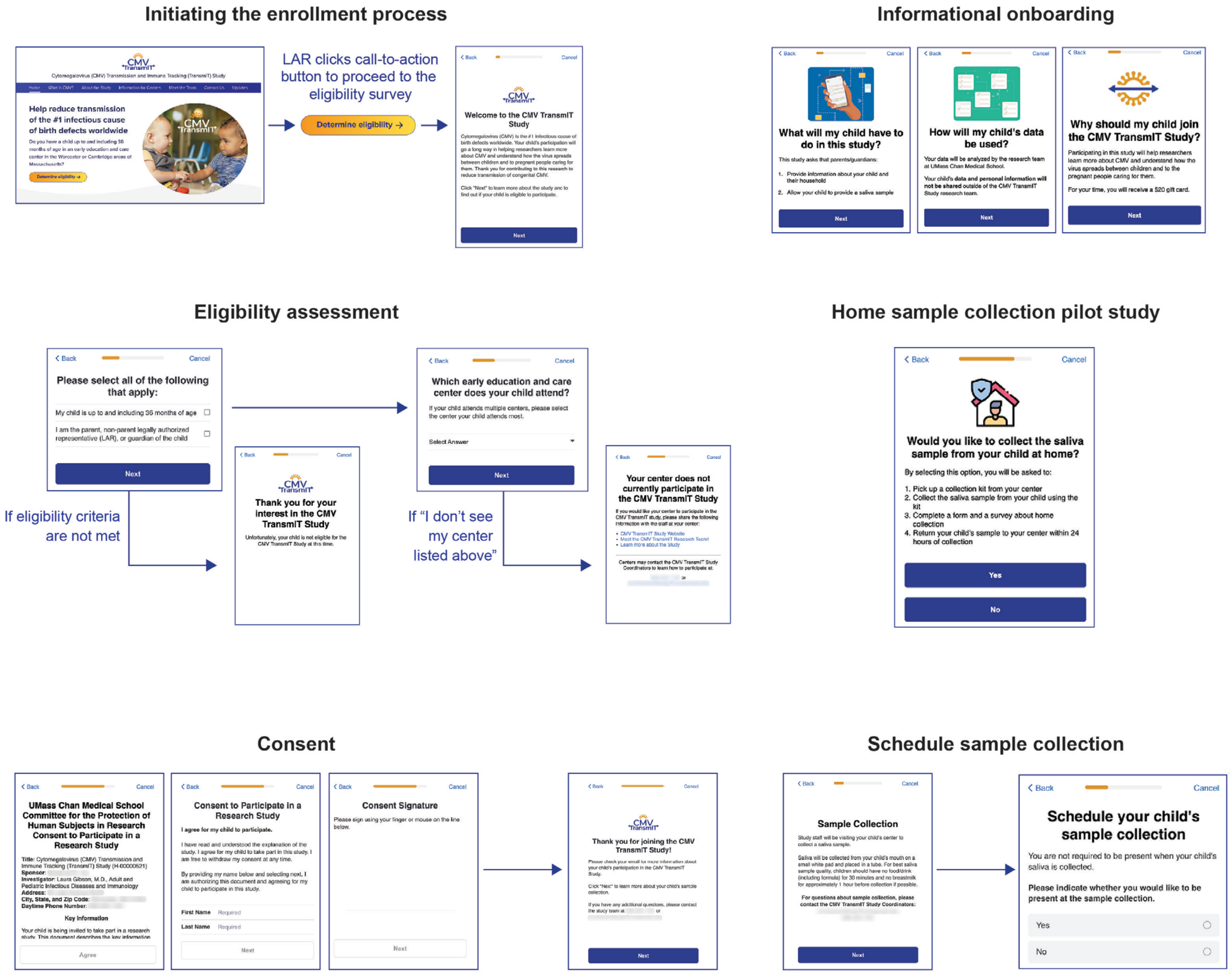
Initiating the enrollment process
Informational onboarding
LAR clicks call-to-action button to proceed to the eligibility survey
Determine eligibility
Help reduce transmission of the #1 infectious cause of birth defects worldwide
Welcome to the CMV TransmIT Study
What will my child have to do in this study?
How will my child's data be used?
Why should my child join the CMV TransmIT Study?
Eligibility assessment
Home sample collection pilot study
Please select all of the following that apply:
My child is up to and including 36 months of age
I am the parent, non-parent legally authorized representative (LAR), or guardian of the child
If eligibility criteria are not met
Thank you for your interest in the CMV TransmIT Study
Which early education and care center does your child attend?
If “I don’t see my center listed above”
Your center does not currently participate in the CMV TransmIT Study
Would you like to collect the saliva sample from your child at home?
By selecting this option, you will be asked to:
1. Pick up a collection kit from your center
2. Collect the saliva sample from your child using the kit
3. Complete a form and a survey about home collection
4. Return your child's sample to your center within 24 hours of collection
Yes
No
Consent
Schedule sample collection
UMass Chan Medical School Committee for the Protection of Human Subjects in Research Consent to Participate in a Research Study
Consent to Participate in a Research Study
Consent Signature
Thank you for joining the CMV TransmIT Study!
Sample Collection
Schedule your child's sample collection
You are not required to be present when your child's saliva is collected.
Please indicate whether you would like to be present at the sample collection.
Back
Cancel
Next
Agree

(C)

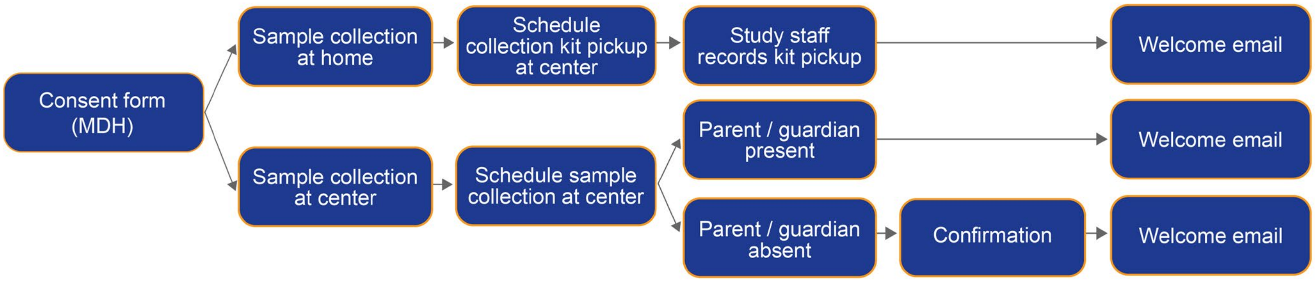

Consent form (MDH)
Sample collection at home
Schedule collection kit pickup at center
Study staff records kit pickup
Welcome email
Sample collection at center
Schedule sample collection at center
Parent / guardian present
Welcome email
Parent / guardian absent
Confirmation
Welcome email

Appendix 3. Communications and social media analytics. (**A**) Top industries, media types and geographic locations associated with press releases reporting the CMV TransmIT Study announcement and enrollment launch. (**B**) Social media engagement between January 1 and November 30, 2024. In June 2022, social media presence for the study was established on Instagram and X (formerly Twitter). Engagement (including likes, retweets, quoted tweets, and replies) with the CMV TransmIT Study was compared with the healthcare/wellness industry. (**C**) Media engagement from *The Conversation* between May 2023 and December 2024.

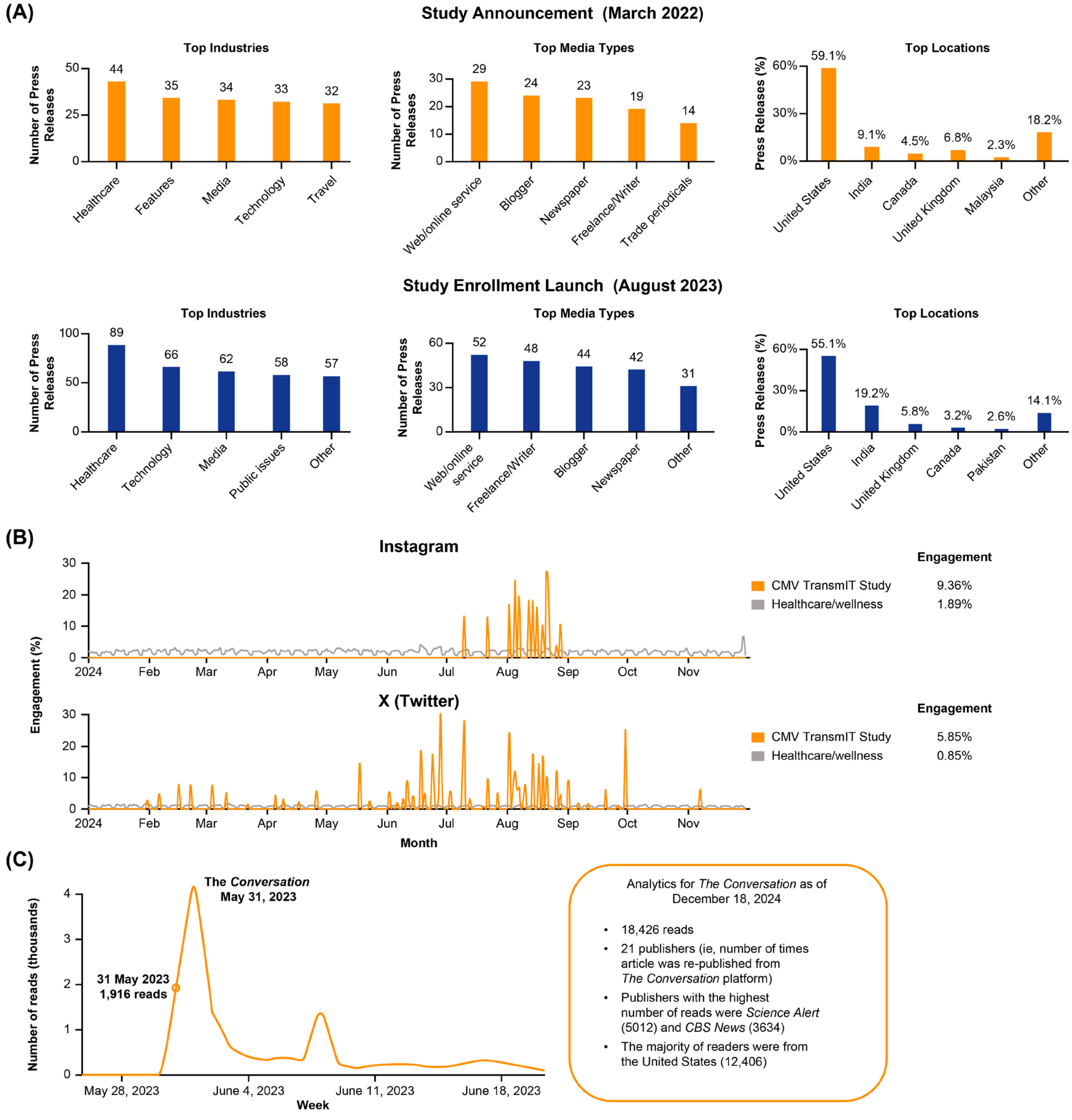

Appendix 4. Early education and care center orientation materials. **(A)** Welcome letter. **(B)** Operations checklist. **(C)** UMass/EEC Collaboration agreement.

(A)

WELCOME

CMV TransmIT

to the Early Education and Care Center Research Network of the CMV Transmission and Immune Tracking Study

A message from Principal Investigator Dr. Laura Gibson

Welcome to the Cytomegalovirus (CMV) Transmission and Immune Tracking (TransmIT) Study Early Education and Care (EEC) Center Research Network! I am so pleased you have decided to partner with me and my study team.

This research project will study CMV transmission in EEC centers in the greater Worcester, MetroWest, and Cambridge/Boston MA areas.

Your participation will make a significant contribution as we seek to understand how CMV spreads among young children and caretakers in EEC centers, In the longer term, we hope this work will help to decrease CMV spread to pregnant caretakers at home or work and to lower the risk of congenital CMV (cCMV).

I invite you to review the attached materials and visit our website to learn more about CMV and our study activities.

My team is excited to visit your center and interact with staff and families. This welcome packet includes a checklist for us to start planning for that visit.

Thank you for joining the CMV TransmIT Study EEC center research network. We look forward to collaborating with you on this study.

EEC Center Research Network Membership

We would like centers to benefit from collaborating with us.
As part of the network, your center will:

- Support health improvement for children and families
- Participate in a community research project
- Receive
  - CMV education for center staff and families
  - Opportunities for networking with other EEC centers
  - Webinars on topics of interest
  - Quarterly newsletter with study updates
  - Annual appreciation gift card
  - Access to a thought-leader in the CMV field and a resource for Infectious Disease issues at your center

CONTACT US
508-523-1181
cmvtransmitstudy@umassmed.edu

VISIT THE STUDY WEBSITE
cmvtransmitstudy.org

(B)

Operations Checklist

Thank you for collaborating with us on the CMV TransmIT Study!

This checklist is used at the operations meeting to clarify study activities and minimize disruption to your center. The information gathered will be used to develop a customized operations plan for your center. If time during or at the end of this meeting, we can develop a schedule for study activities.

This figure gives an overview of the EEC center experience as part of the network:

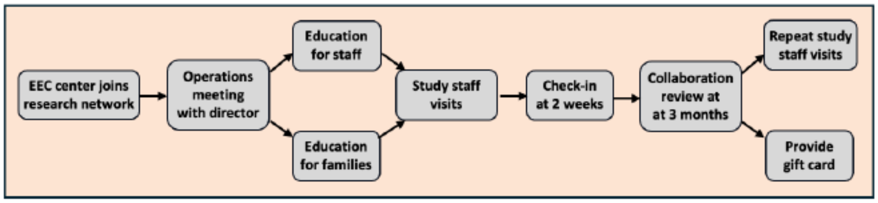

Checklist Items	Notes
Logistics	
How do you prefer we communicate with you?	
Are there any center policies that our staff should know about that might affect our visits? Any safety concerns our staff should be aware of?	
About 2 weeks after study staff visits begin (and as needed), we'll have a check-in meeting to talk about how the study is going.<br><br>After about 3 months, we'll meet again to talk about how our collaboration is going, share feedback, and consider next steps.<br><br>Your center will receive a $300 gift card at that time and every year as appreciation for being part of the network.	
We are asking centers to complete a demographic survey that could take up to 30 minutes to complete. Some of the questions may require a little research or information gathering. The survey will come as a link by email.<br><br>Would you or a staff member be willing to complete the survey?	

Operations Checklist for Centers V4 22AUG2024

Education Sessions	
Our study staff will provide CMV education sessions for families and center staff in-person or via zoom.	
A medical student member of our team is working on an IRB-approved project to test the effectiveness of center staff CMV education sessions.<br><br>Staff are asked to complete a brief knowledge survey before and after session to identify any changes in CMV knowledge. Survey responses are anonymous and no data about center staff is collected.<br><br>Could we send you a survey link to share with your center staff?	
If the parent session is in-person at the center, would the center be able to provide childcare for about 30 minutes?	
Are language translation services needed for staff or families?	
Study Staff Visits	
Study staff visits will be scheduled in advance, which we can do at the end of the meeting if time allows. Activities during study staff visits include meeting families and staff, providing study information, answering questions, offering enrollment to children and center staff, and managing sample collection on site or for participants at home. Other visits can be worked out between our staff and directors as needed (e.g. for sample collection).	
What would be the best place for our staff to set up a table and to collect samples?	Area for meeting families:<br><br>Area for sample collection:
Parents may choose not to be present when their child's sample is collected.<br>In those cases, would a center staff member be able to bring the child to the collection area, identify them to study staff, sit with them during collection, and bring them back to the classroom? The process is expected to take about 5-10 minutes.	
Some participants may choose to collect their sample at home using a study kit that is returned to the center.<br><br>Would a refrigerator be available to store samples until our staff can pick them up?	
What would be the best process for our study staff to engage with center staff and arrange sample collection?	Process to talk with center staff:<br><br>Location for center staff sample collection:

Operations Checklist for Centers V4 22AUG2024

Study Updates and Reporting		
We would like to share study updates with center staff and/or families.<br><br>Could we provide those updates periodically at staff meetings, bulletin boards, emails, or other center communications?		
We will be reporting data and other aspects of the study through scientific journals, research conferences, results summaries, study website, social media, or other academic or public platforms.<br><br>• Do you give us permission to share your center name as part of our research network?<br><br>• Do you give us permission to include your center survey data in anonymous aggregate data (e.g. center survey responses or CMV prevalence)<br><br>• Study staff visits are an opportunity to take pictures of our staff at centers, which may include walls, signage, or other parts of the building but not children or their photographs. Do you give us permission to take these types of pictures?		
Schedules		
Center and study staff identified regular day(s), time(s), duration and designated area(s) for recruitment, enrollment, and sample collection (with or without a parent for children).	Visit date(s) and time(s):	
Center and study staff identified dates and formats for CMV education sessions.	Staff session<br>Date / time:<br><br>Format:	Parent session<br>Date / time:<br><br>Format:
Center and study staff identified dates and formats for check-in and collaboration review meeting.	Check-in meeting<br><br>Date / time:<br><br>Format:	Collaboration review meeting<br>Date / time:<br><br>Format:

(C)

Collaboration Agreement

Between

CMV TransmIT Study at UMass Chan Medical School and

[Insert Center Name]

This agreement documents a collaboration between **[Insert Center Name]** and the CMV TransmIT Study Team in the Department of Medicine, Division of Infectious Diseases and Immunology at UMass Chan Medical School (UMCMS). The study examines how cytomegalovirus (CMV) spreads among young children attending Early Education and Care (EEC) Centers in the Worcester and Cambridge, MA areas. The study is funded by Moderna Therapeutics, Inc.

This collaboration is voluntary. Either partner may choose to withdraw from this agreement by providing thirty (30) days written notice to the other partner and executing a written notice of intent to terminate.

This document aims to describe the background and goals of the study, the general role **of EEC centers** as members of the research network, and the working relationship between **[Insert Center Name]** and the CMV TransmIT Study Team.

DESCRIPTION OF COLLABORATION

Study description

CMV Transmission and Immune Tracking (TransmIT) Study is an observational study to examine CMV transmission and shedding in saliva and urine among young children in large group EEC settings.

Why this study is important

Conducting the study will directly or indirectly contribute to health improvement and CMV prevention in EEC settings and among children and their families.

What we hope to accomplish

The overall project aims to develop a more detailed understanding of CMV prevalence among children, its transmission in EEC settings, and immunologic and virologic factors that reduce viral shedding in children and their household members. To implement the study, we will build a research network of public or private licensed non-home EEC centers in the Cambridge and Worcester, MA areas. Trust, respect, and sustainability will be the foundation of our partnerships with centers. We will regularly seek guidance from the Community Advisory Board on the optimal approach for conducting observational clinical studies in these settings.

Roles and Responsibilities of the EEC Center

- Participate in the CMV TransmIT Study research network of EEC centers. Maintain basic knowledge of the goals and protocol of the study. Inform families about the study and encourage them to review study materials.
- Complete the survey about center demographics and policies. The use of this data will be clarified in the Operations Plan.

- Allow study staff to be at the center for recruitment and enrollment at regularly scheduled times mutually acceptable to the center and to study staff. Identify a location(s) within the center where study staff can reasonably talk with families and collect samples.
- If a parent declines to be present when samples are collected from their child, then complete and inform study staff about additional requirements if any (e.g. parent permission slip), transport the child to the collection area, identify them to study staff, hold or comfort or otherwise assist the child as needed, and transport them from the collection area after samples are obtained. The details of this process will be clarified in the Operations Plan.
- Engage in bi-directional communication throughout the study, including any feedback and mutually agreeable operational changes that optimize both relationships and study implementation.
- Participate in a mutual re-evaluation of the relationship after an initial 3-month trial period then regular (at least yearly) intervals, at which points center and study leadership will confirm or decline to continue the relationship.

Roles and Responsibilities of the CMV TransmIT Study Team

- Develop an Operations Plan in collaboration with **[Insert Center Name]** leadership that minimizes disruption and inconvenience for children, families, and center staff. The plan will include clarifying any use of the center name and/or data in publications or public forums (e.g., study website or newsletter). Action items pending and completed will be tracked in the Operations Checklist. All operations documents will be shared with the center.
- Adhere to center policies. Become familiar with and adapt to center routines. Minimize inconvenience and burden to centers. Seek ways to help with study and center workflows.
- Share CMV educational resources and study information with center leadership and staff.
- Visit the center at least once after the study launches to meet families, introduce study staff, share educational resources, and study information, and answer questions in a location(s) acceptable to the center as above. Study materials will prominently display the contact information of study staff for parents to obtain more information or ask questions. No study enrollment will occur at this visit(s).
- Maintain the confidentiality of participants and study data. Only aggregate data relating to CMV at the center will be shared with centers. Data will not identify or link to individual participants or their families will not be identified, and centers will not be informed which children are enrolled. Do not share data with unauthorized third parties.
- Engage in bi-directional communication throughout the study, including any feedback and mutually agreeable operational changes that optimize both relationships and implementation of the study. Remain accessible and responsive to centers for any questions or problems. Provide regular study updates.
- Participate in a 3-month mutual trial period after which the relationship will be re-evaluated. At the end of the 3-month trial period, a $300 gift card will be offered to **[Insert Center Name]** to thank them for participating in the research network. If both partners continue to work together after one year, then another gift card up to $300 will be offered every year.

General Provisions

- The CMV TransmIT Study at UMass Chan Medical School and **[Insert Center Name]** are separate and independent entities, and this agreement shall not create an employer/employee or joint employer relationship between them.
- This agreement shall not create any financial obligations between the CMV TransmIT Study at UMass Chan Medical School and **[Insert Center Name]**. In no event shall either be liable to the other for any indirect, incidental, or consequential damage of any nature or kind resulting from or arising in connection with this agreement.
- This agreement shall be governed by the laws of the Commonwealth of Massachusetts. The CMV TransmIT Study at UMass Chan Medical School and **[Insert Center Name]** agree that the state courts of the Commonwealth of Massachusetts shall have exclusive jurisdiction over any action arising out of or relating to this document.
- This document may only be modified or amended by the mutual written agreement of the CMV TransmIT Study at UMass Chan Medical School and **[Insert Center Name]** and executed by their authorized representatives.

The undersigned acknowledge and agree to this collaboration, which takes effect on the date below.

**[Insert center name]**	CMV TransmIT Study
**[Insert center city]**	UMass Chan Medical School
______________________________	______________________________
**[Insert representative name]**	Laura Gibson, M.D.
**[Insert representative title or role]**	Principal Investigator
Date: ___/___/___	Date: ___/___/___

Appendix 5. Early education and care center survey.

CMV Transmission and Immune Tracking Study<br>Center Survey Questions for use in REDCap platform	
**Staff**	
How many total staff **regularly** work at your center in any role?<br>Include all employed, contracted, volunteer, and full or part time.<br>Do not include temporary workers such as trainees or substitute teachers.	[Enter number in text field]
Of the total staff, how many spend **most** of their time in the classroom with children?<br>Include all employed, contracted, volunteer, and full or part time.<br>Do not include temporary workers such as trainees or substitute teachers.	
**Enrollment**	
What is the total number of children enrolled at your center?	___[Enter number in text field]
Does your center use the Massachusetts EEC Child Age Classifications?<br>Infants (up to 15 months)<br>Toddlers (15 to 33 months)<br>Preschoolers (33 months to school age)	Yes<br>No
If NO: How do you classify their ages? Please list name and age range of each category.	___[Enter number in text field]
How many **total slots** do you have at your center for children **ages 36 months or less**?	___[Enter number in text field]
How many children are **currently enrolled** in these slots?	___[Enter number in text field]
About how many new children enroll at your center every year?	___[Enter number in text field]
About how many new children **ages 36 months or less** enroll at your center every year?	___[Enter number in text field]
How many days per week is your center open?	[select number 1-7 dropdown]
For ALL: Approximate percentage of children who are **only** at the center for 1 day a week	___[Enter number in text field]
For 2 days: Approximate percentage of children who are **only** at the center for 2 days a week	___[Enter number in text field]

Center Survey V6 STUDY00000521 02OCT2024

For 3 days: Approximate percentage of children who are only at the center for 3 days a week	___[Enter number in text field]
For 4 days: Approximate percentage of children who are only at the center for 4 days a week	___[Enter number in text field]
For 5 days: Approximate percentage of children who are only at the center for 5 days a week	___[Enter number in text field]
For 6 days: Approximate percentage of children who are only at the center for 6 days a week	___[Enter number in text field]
For 7 days: Approximate percentage of children who are only at the center for 7 days a week	___[Enter number in text field]
**Demographics**	
What is the approximate percentage of **all children** at your center in the following racial or ethnic categories?	___[Enter number in text field for each]<br>White<br>Black or African American<br>Hispanic or Latino/a<br>Asian<br>Native American<br>Pacific Islander / Native Hawaiian<br>Multiracial
What are the zip codes where all families at your center live (not where they are when their child is at the center e.g. workplace)? Please enter each one into a field shown. If you need more fields, they will be shown after the last field here.	[Matrix of text fields for zip codes]
**Financial Assistance for Families**	
What percentage of families are using government assistance **to attend your** center?	___[Enter number in text field]
What government assistance programs are your families using? Check all that apply.	Child Care Development Fund (CCDF) voucher<br>Tax Credits<br>Mass.gov Programs<br>Tuition Assistance<br>Reduced Cost Programs<br>Transitional Aid<br>TEFAP<br>WIC<br>Other, please specify ______________________________<br>Don't know<br>Prefer not to say
**Infection Control**	

Does your center have a document, website, or other written resource outlining policies and procedures for infection control?	Yes<br>No<br>Don't know<br>Prefer not to say
If YES: Would you be willing to share that resource with us? It would only be used by us and not shared with any<br>outside entity.	Yes<br>No<br>Don't know<br>Prefer not to say
If YES SHARE: Please attach your infection control resource here. For the questions below, you can just add any information that is not already in that resource.	[File Upload]<br>[Paste link]
How Does your center train staff about infection control? Check all that apply members about infection control? Check all that apply	Live presentation (in person or<br>remote) Self-directed with<br>recorded presentation Self-<br>directed with written<br>information Live presentation<br>and self-directed with recorded<br>presentation Self-directed with<br>written information Live<br>presentation and self-directed<br>Center does not specifically train about infection control<br>Other, please specify<br>Don't know<br>Prefer not to say
How does your center confirm that staff members have a sufficient understanding of infection control?	Written test<br>Oral test<br>Upon completion of electronic training<br>Direct observation while working<br>Center does not specifically confirm understanding of infection control<br>Other, please specify ___________________________<br>Don't know<br>Prefer not to say
Does your center prohibit children from sharing certain items?	Yes<br>No<br>Don't know<br>Prefer not to say
If YES: Which items? Check all that apply:	Food<br>Drinks<br>Utensils<br>Dishes<br>Napkins or other facial cloth<br>Highchairs<br>Toothbrushes<br>Linens (e.g. towels or blankets)<br>Sleeping mats

	Other, please specify ________________________________<br>Don't know<br>Prefer not to say
Does your center specify the **situations** in which staff must wash their hands?	Yes<br>No<br>Don't know<br>Prefer not to say
If YES: What are the situations? Please list	[text]
Does your center specify **how many times per day** staff must wash their hands?	Yes<br>No<br>Don't know<br>Prefer not to say
If YES: How many times per day?	___[Enter number in text field]
Does your center specify how often staff should wash surfaces, toys, and equipment per day?	Yes No<br>Don't know<br>Prefer not to say
If YES: How often per day?	___[Enter number in text field]
Does your center specify what cleaning products are used?	Yes No<br>Don't know<br>Prefer not to say
What types of cleaning products are used routinely at your center?	Dish or hand soap (liquid or foam)<br>Bleach-based cleaners<br>Vinegar-based cleaners<br>Other, please specify ________________________________<br>Don't know<br>Prefer not to say

Which immunizations are required for enrollment at your center? Check all that apply	Hepatitis A<br>Hepatitis B<br>Rotavirus<br>Diphtheria, tetanus, and acellular pertussis (DTaP)<br>Haemophilus Influenza type B (Hib)<br>Pneumococcus<br>Inactivated polio virus (IPV)<br>Influenza<br>COVID-19<br>Measles, mumps and rubella (MMR)<br>Varicella<br>Other, please specify<br>No specific immunizations are required<br>Don't know<br>Prefer not to say
Does your center have a specific isolation area for children that become sick during the day?	Yes<br>No<br>Don't know<br>Prefer not to say
If YES: Where is the area?	In a room or other area with other children<br>In a room or other area separate from other children<br>Other, please specify<br>Don't know<br>Prefer not to say
If NO: Where are sick children typically located until they leave the center?	In a room or other area with other children<br>In a room or other area separate from other children<br>Other, please specify<br>Don't know<br>Prefer not to say
For BOTH: Do you have any additional comments about infection control at your center?	[text]
**Daily Activities**	
How many classrooms are specifically for **infants**?	[Enter number in text field]
How many classrooms are specifically for **toddlers**?	[Enter number in text field]
How many classrooms are specifically for **preschoolers**?	[Enter number in text field]
How many separate rooms do **infants** typically spend time in during the day?	___[Enter number in text field]
How many separate rooms do **toddlers** typically spend time in during the day?	___[Enter number in text field]

How many separate rooms do **preschoolers** typically spend time in during the day?	___[Enter number in text field]
Does your center have dedicated unstructured or open **play time** during the day when children can interact?	Yes<br>No<br>Don't know<br>Prefer not to say
If YES: How many times per day?	___[Enter number in text field]
If YES: How long do play periods typically last (in minutes)?	___[Enter number in text field]
Does your center have specific **meal or snack times** for feeding children during the day?	Yes<br>No<br>Don't know<br>Prefer not to say
If YES: How many times per day?	___[Enter number in text field]
If YES: How long do meal or snack times typically last (in minutes)?	___[Enter number in text field]
If NO: When is feeding typically done? Check all that apply.	Whenever each child needs or requests feeding<br>When enough staff is available for feeding in each classroom<br>Any time food is delivered during the day<br>Other, please specify<br>Don't know<br>Prefer not to say
Does your center have a specific **location(s)** for feeding children?	Yes No<br>Don't know<br>Prefer not to say
If YES: Where is the feeding location(s)? Check all that apply.	In a room or other area with other children<br>In a room or other area separate from other children<br>In a room or other area with play, sleep, or other non-food areas<br>In a room or other area separate from play, sleep, or other non-food areas<br>Other, please specify ____________<br>Don't know<br>Prefer not to say

If YES: How often is the feeding location(s) cleaned?	After every use<br>One time daily<br>More than one time daily<br>Every other day<br>Weekly<br>No specific frequency<br>Other, please specify ______________<br>Don't know<br>Prefer not to say
If NO: Where is feeding typically done? Check all that apply.	In a room or other area with other children<br>In a room or other area separate from other children<br>In a room or other area with play, sleep, or other non-food areas<br>In a room or other area separate from play, sleep, or other non-food areas<br>Other, please specify ______________<br>Don't know<br>Prefer not to say
What is the approximate percentage of **infants** that are **spoon fed** by staff?	___[Enter number in text field] spoon fed by staff
What is the approximate percentage of **infants** that are **bottle fed** by staff?	___[Enter number in text field] bottle fed by staff
What is the approximate percentage of **infants** that are self-fed?	___[Enter number in text field] self-fed
What is the approximate percentage of **toddlers** that are **spoon fed** by staff?	___[Enter number in text field]
What is the approximate percentage of **toddlers** that are **bottle fed** by staff?	___[Enter number in text field]
What is the approximate percentage of **toddlers** that are **self-fed?**	___[Enter number in text field]
What is the approximate percentage of **preschoolers** that are **spoon fed** by staff?	___[Enter number in text field]
What is the approximate percentage of **preschoolers** that are **bottle fed** by staff?	___[Enter number in text field]
What is the approximate percentage of **preschoolers** that are **self-fed?**	___[Enter number in text field]
Does your center have a specific location(s) for changing diapers?	Yes<br>No<br>Don't know<br>Prefer not to say

IF YES, where is the diapering location(s)?	In a room or other area with other children<br>In a room or other area separate from other children<br>Other, please specify _____________<br>Don't know<br>Prefer not to say
IF YES, how often is the diapering location(s) cleaned?	After every use<br>One time daily<br>More than one time daily<br>Every other day<br>Weekly<br>No specific frequency<br>Other, please specify _____________<br>Don't know<br>Prefer not to say
IF NO, where is diapering typically done?	In a room or other area with other children<br>In a room or other area separate from other children<br>Wherever space is available (e.g. floor)<br>Other, please specify _____________<br>Don't know<br>Prefer not to say
Does your center have a specific location(s) for toileting children?	Yes<br>No<br>Don't know<br>Prefer not to say
IF YES, where is the toileting location(s)?	In a room or other area with other children<br>In a room or other area separate from other children<br>Other, please specify _____________<br>Don't know<br>Prefer not to say
IF YES, how often is the toileting location(s) cleaned?	After every use<br>One time daily<br>More than one time daily<br>Every other day<br>Weekly<br>No specific frequency<br>Other, please specify _____________<br>Don't know<br>Prefer not to say
IF NO, where is toileting typically done?	In a room or other area with other children<br>In a room or other area separate from other children<br>Other, please specify _____________<br>Don't know<br>Prefer not to say
Do you have any additional comments about these daily activities at your center?	[enter text]

Local Community	
We would like to get to know the communities around the centers in our research network. If applicable, please share details about locations and people as below or any others you would suggest.	
Public places or activities that are popular in your catchment area, such as community centers, festivals, fundraisers, or clothing/food drives	[Enter text]
Trusted community leaders that your families tend to connect with, such as houses of worship, advocates, or neighborhood representatives.	[Enter text]

Appendix 6. Quarterly newsletter.

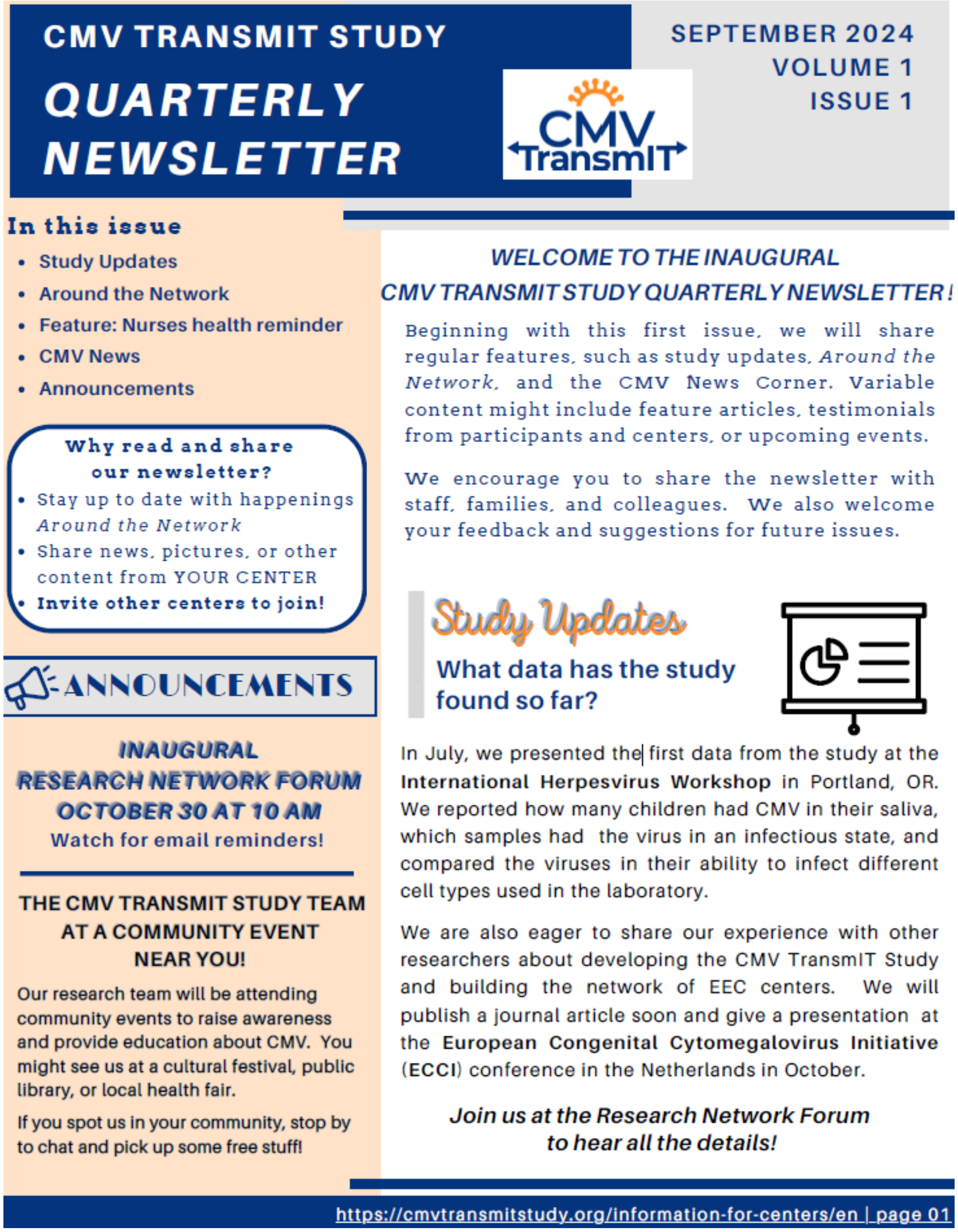

CMV TRANSMIT STUDY

QUARTERLY NEWSLETTER

SEPTEMBER 2024
VOLUME 1
ISSUE 1

CMV TransmIT

In this issue

- Study Updates
- Around the Network
- Feature: Nurses health reminder
- CMV News
- Announcements

Why read and share our newsletter?

- Stay up to date with happenings *Around the Network*
- Share news, pictures, or other content from YOUR CENTER
- **Invite other centers to join!**

ANNOUNCEMENTS

INAUGURAL RESEARCH NETWORK FORUM OCTOBER 30 AT 10 AM

Watch for email reminders!

THE CMV TRANSMIT STUDY TEAM AT A COMMUNITY EVENT NEAR YOU!

Our research team will be attending community events to raise awareness and provide education about CMV. You might see us at a cultural festival, public library, or local health fair.

If you spot us in your community, stop by to chat and pick up some free stuff!

WELCOME TO THE INAUGURAL CMV TRANSMIT STUDY QUARTERLY NEWSLETTER!

Beginning with this first issue, we will share regular features, such as study updates, *Around the Network*, and the CMV News Corner. Variable content might include feature articles, testimonials from participants and centers, or upcoming events.

We encourage you to share the newsletter with staff, families, and colleagues. We also welcome your feedback and suggestions for future issues.

Study Updates

What data has the study found so far?

In July, we presented the first data from the study at the **International Herpesvirus Workshop** in Portland, OR. We reported how many children had CMV in their saliva, which samples had the virus in an infectious state, and compared the viruses in their ability to infect different cell types used in the laboratory.

We are also eager to share our experience with other researchers about developing the CMV TransmIT Study and building the network of EEC centers. We will publish a journal article soon and give a presentation at the **European Congenital Cytomegalovirus Initiative** (**ECCI**) conference in the Netherlands in October.

Join us at the Research Network Forum to hear all the details!

https://cmvtransmitstudy.org/information-for-centers/en | page 01

Note: Only the first page of this document is included here for illustrative purposes. The complete version is available upon request.

Appendix 7. Study participant recruitment materials. **(A)** CMV fact sheet. **(B)** Study FAQs. **(C)** Recruitment poster.

(A)

What You Should Know about CMV

What is cytomegalovirus? [1]

- The name is pronounced **"sy-toe-MEG-a-low-vy-rus"** but "CMV" is typically used.
- CMV is a **common infection** in the same family of viruses as chicken pox and cold sores. Viruses in this family stay in the body for life.
- Infection can occur at any age.
- Most people have **mild or no symptoms** and **no long- term health effects.**

How does CMV spread?

- The virus spreads mainly through **saliva and urine.**
- Children in large group programs can get CMV from each other. They have lots of virus in their saliva and urine for a long time after infection even though they look healthy.
- **The virus can also spread from children to adult caretakers** through contact with saliva or urine during activities such as feeding or changing diapers.

If caretakers are PREGNANT, CMV can spread to their developing baby

- Infection of a baby before birth is called **congenital CMV (cCMV).**[1,2]
- Transmission to the fetus occurs in about 2 of every 5 people who get CMV for the first time during pregnancy.
- CMV is the **most common cause of birth defects in the U.S.**[3] and affects about **1 of every 200 babies born each year.**
- By comparison, Down syndrome is the next most common cause[3] and affects about 1 of every 700 babies born each year.

What health effects can cCMV cause?

- Most babies with cCMV never have health problems from the virus.
- For some babies, symptoms can be **seen at birth** (such as small head size or yellow skin) or **appear or worsen later** in childhood (such as cerebral palsy or developmental delays).
- The most common health effect is **hearing loss.**
- About 1 of every 5 babies with cCMV have **lifelong disabilities.**

To lower YOUR risk of getting CMV, especially during pregnancy, *avoid direct contact with saliva and urine*

Here are some examples:

- Avoid kissing children near the mouth or nose.
- Do not put a pacifier or toothbrush used by a child into your mouth.
- Avoid sharing utensils, dishes, food, drinks, or straws with children.
- Wash your hands after feeding, diapering/toileting, or cleaning children. *Use soap and water for at least 20 seconds.*
- Disinfect toys, surfaces, or other objects that might have contacted a child's saliva or urine.
- People who have contact with children should treat all body fluids as if they are infectious.

References
1. https://www.cdc.gov/cmv/index.html;
2. https://www.nationalcmv.org/
3. https://www.cdc.gov/ncbddd/birthdefects/index.html

UMass Chan MEDICAL SCHOOL

Find out more about CMV at the Centers for Disease Control and Prevention (CDC) website

CMV TransmIT

Fact Sheet V3 IRB00000521 02OCT2024

(B)

Cytomegalovirus (CMV) Transmission and Immune Tracking (TransmIT) Study

Research Happening at Your Center

What is Cytomegalovirus?

- Cytomegalovirus (CMV) is a common virus that spreads mostly through **saliva and urine.**
- Most people don't get very sick or have any long-term health effects from the virus.
- Young children in large groups can get CMV from each other.
- The virus can **spread from children to adult caretakers** through saliva or urine during activities such as feeding or diaper changing.

Why is this virus a problem?

- If caretakers are **PREGNANT**, CMV can spread to their developing baby.
- Infection of a baby before birth is called **congenital CMV (cCMV)**.
- CMV is the **most common cause of birth defects** in the U.S. and affects about **1 of every 200 babies** born each year.
- About 1 of every 5 babies with cCMV have **lifelong disabilities**.

What is this research about?

- Researchers want to study how CMV spreads in early education and care centers and how the body fights CMV in saliva and urine.
- Results of the study may help decrease CMV spread from children to their pregnant caretakers at home or work.
- Decreasing CMV spread to pregnant people could also **lower the risk of cCMV**.

How can I learn more or enroll in the study?

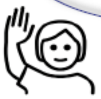

Contact us

508-523-1181

cmvtransmitstudy@umassmed.edu

CMV TransmIT

Visit the study website

< insert QR code >

www.cmvtransmitstudy.org

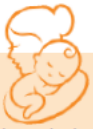

How can I help?

- Enroll in the CMV TransmIT Study to help researchers understand CMV spread in childcare centers.

What does the study involve?

- Participants fill out an online survey and provide a saliva sample for a CMV test. Saliva can be obtained at the center or at home using a small sponge on a swab.
- Time commitment is expected to be about 30 minutes total.

Do I get the CMV test result?

- People who enroll in the study will learn about CMV testing and results in their consent form.

What do I get for participating?

- You will receive a $20 gift card for your time.

A research collaboration between UMass Chan MEDICAL SCHOOL *and* moderna *to study how CMV spreads*

FAQ V3 IRB00000521 02OCT2024

(C)

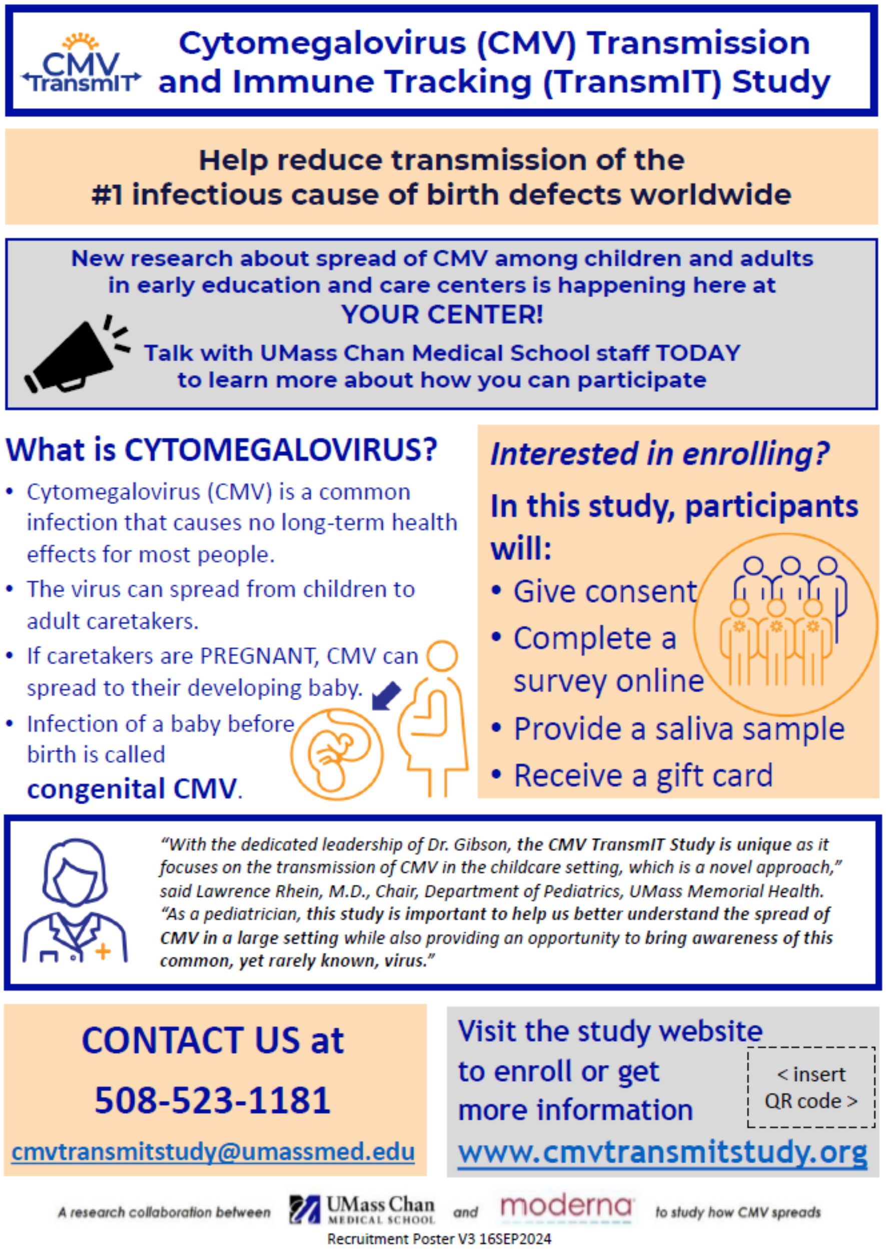
CMV TransmIT
Cytomegalovirus (CMV) Transmission and Immune Tracking (TransmIT) Study
Help reduce transmission of the #1 infectious cause of birth defects worldwide
New research about spread of CMV among children and adults in early education and care centers is happening here at
YOUR CENTER!
Talk with UMass Chan Medical School staff TODAY to learn more about how you can participate
What is CYTOMEGALOVIRUS?
• Cytomegalovirus (CMV) is a common infection that causes no long-term health effects for most people.
• The virus can spread from children to adult caretakers.
• If caretakers are PREGNANT, CMV can spread to their developing baby.
• Infection of a baby before birth is called congenital CMV.
Interested in enrolling?
In this study, participants will:
• Give consent
• Complete a survey online
• Provide a saliva sample
• Receive a gift card
"With the dedicated leadership of Dr. Gibson, the CMV TransmIT Study is unique as it focuses on the transmission of CMV in the childcare setting, which is a novel approach," said Lawrence Rhein, M.D., Chair, Department of Pediatrics, UMass Memorial Health. "As a pediatrician, this study is important to help us better understand the spread of CMV in a large setting while also providing an opportunity to bring awareness of this common, yet rarely known, virus."
CONTACT US at
508-523-1181
cmvtransmitstudy@umassmed.edu
Visit the study website to enroll or get more information
< insert QR code >
www.cmvtransmitstudy.org
A research collaboration between UMass Chan Medical School and moderna to study how CMV spreads
Recruitment Poster V3 16SEP2024

Appendix 8. Participant surveys. **(A)** Children. **(B)** Early education and care center staff.

(A)

CMV Transmission and Immune Tracking Study<br>Child Participant Survey Questions for use in MyDataHelps platform	
**Eligibility**	
What is the child's birthdate?	Enter MM/DD/YYYY
What is your relationship with the child?	Parent<br>Non-parent Legally Authorized Representative (LAR) or guardian<br>Other
**Demographics**	
What sex was the child assigned at birth on their birth certificate?	Male<br>Female<br>Intersex<br>Prefer not to say<br>Don't know
What is the child's race? Check all that apply	White<br>Asian<br>African American<br>Pacific Islander/Native Hawaiian<br>Native American<br>Other Please specify<br>Prefer not to say<br>Don't know
Is the child Hispanic or Latinx?	Yes<br>No<br>Prefer not to say<br>Don't know
When did your child start at the center? If you're not sure, enter an approximate date.	[Enter date in calendar]
**Caretakers**	
**For questions in this section, "primary caretaker" is defined as the person who *lives in the home* and *on average does more than half* of the weekly care needed by the child. This person may or may not be a parent or Legally Authorized Representative.**	
Are you the primary caretaker of the child?	Yes<br>No<br>Prefer not to say<br>Don't know
What relationship does the primary caretaker have with the child?	Female biological parent<br>Male biological parent<br>Non-parent Legally Authorized Representative (LAR) or guardian<br>Non-parent family member (such as a household member)

Participant Survey for Children Stage I_V3_IRB00000521_04MAR2024 1

	Other<br>Prefer not to say<br>Don't know
What is the age of the **primary caretaker**?	___[Enter number in text field]<br>Prefer not to say<br>Don't know
What is the age of the **female biological parent**?	___[Enter number in text field]<br>Prefer not to say<br>Don't know
What is the age of the **male biological parent**?	___[Enter number in text field]<br>Prefer not to say<br>Don't know
What language is the **primary caretaker** most comfortable speaking	English<br>Spanish<br>English and Spanish<br>Other Please specify<br>Other and English Please specify<br>Prefer not to say<br>Don't know
Does the child live with anyone identifying as LGBTQ+?	Yes<br>No<br>Prefer not to say<br>Don't know
IF YES, Does the **primary caretaker** identify as LGBTQ+	Yes<br>No<br>Prefer not to say<br>Don't know
What is the marital status of the **primary caretaker**?	Married and/or living with a partner (common law)<br>Divorced<br>Separated<br>Widowed (not living common law)<br>Single<br>Never married (not living common law)<br>Prefer not to say<br>Don't know
Which of the following best describes the highest level of education completed by the **primary caretaker**?	Some high school or technical/vocational or other training program<br><br>Graduated from a 2-year technical/vocational or other training program<br><br>Graduated from a 4-year high school or technical/vocational program or completed GED (General Education Diploma)

	Less than 2 years of college<br><br>2 years or more of college including associate degree or equivalent<br><br>Graduated from college<br><br>Master's degree (or other post-graduate training)<br><br>Doctoral degree (such as PhD., MD, EdD, DVM, DDS, or JD)<br><br>Prefer not to say<br><br>Don't know
Which of the following best describes the highest level of education completed by a **biological parent**?	Some high school or technical/vocational or other training program<br><br>Graduated from a 2-year technical/vocational or other training program<br><br>Graduated from a 4-year high school or technical/vocational program or completed GED (General Education Diploma)<br><br>Less than 2 years of college<br><br>2 years or more of college including associate degree or equivalent<br><br>Graduated from college<br><br>Master's degree (or other post-graduate training)<br><br>Doctoral degree (such as PhD, MD, EdD, DVM, DDS, or JD)<br><br>Prefer not to say<br><br>Don't know
**Household**	
In how many households does the child spend a large amount of time (such as 2 nights per week)?	Number entry from dropdown (1, 2, 3, 4, 5 or more)<br>Prefer not to say<br>Don't know
IF OTHER THAN 1 approximately how much time is spent at each household per week (in percent of 1 week)?	Household 1 (primary) ___[Enter number in text field]<br>Household 2 ___[Enter number in text field]<br>Household 3 ___[Enter number in text field]<br>Household 4 ___[Enter number in text field]<br>Any other households - ___[Enter number in text field]<br>Prefer not to say<br>Don't know

Are you a household member of the child?	Yes, the primary household<br>Yes, a non-primary household<br>No<br>Prefer not to say<br>Don't know
How many adults (18 years and older **including yourself** if applicable) are permanent residents of the child's primary household? These terms refer to the home that you or other adults consider to be the place or address where they live.	___[Enter number in text field]<br>Prefer not to say<br>Don't know
How many children (less than 18 years) are permanent residents of the child's primary household?	___[Enter number in text field]<br>Prefer not to say<br>Don't know
What languages are spoken by most members of the child's primary household?	English<br>Spanish<br>Other Please specify
**In the past year**, have you and/or other financially responsible adult(s) been almost or completely unable to pay for the child's expenses?	Yes<br>No<br>Prefer not to say<br>Don't know
**In the past year**, has the child's primary household had difficulty accessing any of the following? Check all that apply.	Food<br>Heat<br>Running water<br>Electricity<br>Clothing<br>Medical care<br>Medication<br>Other<br>Prefer not to say<br>Don't know
Does anyone in the child's primary household have income-based government assistance of any kind?	Yes<br>No<br>Prefer not to say<br>Don't know
What is the child's primary form of health insurance?	Insurance through a current or former employer or union (yours or another family member's). This includes COBRA.<br><br>Insurance purchased directly from an insurance company (by you or another family member). This includes coverage purchased through an exchange or marketplace, such as HealthCare.gov.

	Any kind of state or government-sponsored assistance plan based on income or disability<br>IF YES →<br>Medicare for people 65 and older or people with certain disabilities.<br>Medicaid<br>Other Please specify<br><br>TRICARE or other military health care, including VA health care.<br><br>Indian Health Service.<br><br>No insurance coverage<br><br>Prefer not to say<br><br>Don't know
Please indicate how often you or other adults in the child's household participate in the behaviors listed	Kiss child on lips or cheek [enter Never, Rarely, Sometimes, Often, Always]<br><br>Share food, utensils, drinks, straws, pacifiers, or toothbrushes with the child [enter Never, Rarely, Sometimes, Often, Always]<br><br>Wash hands after changing diapers, wiping child's face, feeding child, or handling toys [enter Never, Rarely, Sometimes, Often, Always]<br><br>Disinfect toys, countertops, and other surfaces that might come in contact with a child's saliva or urine [enter Never, Rarely, Sometimes, Often, Always]
Are there any cultural, religious, or other practices in the child's household(s) that may involve contact with the child's saliva or urine?	Yes, Please describe<br>No<br>Prefer not to say<br>Don't know
**Medical**	
**In the past 14 days**, has the child had any of these symptoms? Check all that apply	Cough, runny nose, sore throat, trouble breathing, wheezing or other respiratory<br>Vomiting, diarrhea, belly pain, or other intestinal<br>Headache, joint, or other pain<br>Rash or other skin<br>Fever or other signs of infection<br>General symptoms such as fatigue, low appetite, muscle aches, or irritability<br>Other<br>None<br>Prefer not to say<br>Don't know

Has the child **ever** been diagnosed with wheezing, asthma, or any other lung or breathing condition?	Yes<br>No<br>Prefer not to say<br>Don't know
***IF YES,** has* the child been diagnosed **in the past 14 days**?	Yes<br>No<br>Prefer not to say<br>Don't know
Has the child **ever** been hospitalized?	Yes<br>No<br>Prefer not to say<br>Don't know
***IF YES,* In the past 6 months**, how many times was the child hospitalized?	___[Enter number in text field]<br>Prefer not to say<br>Don't know
IF YES, for what type of problem(s) was the child hospitalized? Check all that apply	Cough, runny nose, sore throat, sinuses, ear pain, or other symptoms of a cold<br>Trouble breathing, wheezing or other symptoms in the chest<br>Vomiting, diarrhea, stomach pain, or other belly symptoms<br>Headache, joint, or other pain<br>Rash or other skin<br>Fever or other signs of infection<br>General symptoms such as fatigue, low appetite, muscle aches, or irritability<br>Other<br>Prefer not to say<br>Don't know
Has the child **ever** been diagnosed by a healthcare provider to have a developmental delay? Examples include not crawling or talking on time.	Yes<br>No<br>Prefer not to say<br>Don't know
Has the child **ever** been diagnosed by a healthcare provider to have a low immune system?	Yes<br>No<br>Prefer not to say<br>Don't know
Has the child **ever** been tested for CMV?	Yes<br>No<br>Prefer not to say<br>Don't know
IF YES, when was the test done?	In the past 14 days<br>In the past 6 months

	In the past year<br>More than 1 year ago<br>Prefer not to say<br>Don't know
IF YES, what was the result?	Positive<br>Negative<br>Inconclusive<br>Prefer not to say<br>Don't know
Have any household members of the child **ever** been tested for CMV?	Yes<br>No<br>Prefer not to say<br>Don't know
IF YES, when was the test done?	In the past 14 days<br>In the past 6 months<br>In the past year<br>More than 1 year ago<br>Prefer not to say<br>Don't know
IF YES, what was the result?	Positive<br>Negative<br>Inconclusive<br>Prefer not to say<br>Don't know
Breastfeeding	
Has the child ever received breast milk in any form, such as nursing, bottle/cup, or mixed with formula or food?	Yes<br>No<br>Prefer not to say<br>Don't know
Has the child received breast milk in any form **in the past 14 days**?	Yes<br>No<br>Prefer not to say<br>Don't know
IF YES, on average, how many times did the child receive breast milk **per 24-hour period?**	___[Enter number in text field]<br>Prefer not to say<br>Don't know
**IF YES,** on average, how much breast milk did the child receive **per feeding session**? Check all that apply	Nursing ____minutes [Enter number in text field]<br>Other forms ____ounces [Enter number in text field]<br>Prefer not to say<br>Don't know

(B)

CMV Transmission and Immune Tracking Study<br>Staff Participant Survey Questions for use in REDCap platform	
**Demographics**	
What sex were you assigned at birth on your birth certificate?	Male<br>Female<br>Intersex<br>Prefer not to say<br>Don't know
What is your race? Check all that apply	White<br>Asian<br>African American<br>Pacific Islander/Native Hawaiian<br>Native American<br>Other, Please specify<br>Prefer not to say<br>Don't know
Are you Hispanic or LatinX?	Yes<br>No<br>Prefer not to say<br>Don't know
What language(s) are you most comfortable speaking? Select all that apply	English<br>Spanish<br>Other, Please specify<br>Prefer not to say<br>Don't know
Do you identify as LGBTQ+?	Yes<br>No<br>Unsure<br>Prefer not to say
What is your marital status?	Married and/or living with a partner (common law)<br>Divorced<br>Separated<br>Widowed (not living common law)<br>Single<br>**Never married (not living common law)**<br>Prefer not to say<br>Don't know
Which of the following best describes your **highest level** of education?	Some high school or technical/vocational or other training program<br><br>Graduated from a 2-year technical/vocational or other training program

	Graduated from a 4-year high school or technical/vocational program or completed GED (General Education Diploma)<br><br>Less than 2 years of college<br><br>2 years or more of college including associate degree or equivalent.<br><br>Graduated from college<br><br>Master's degree (or other post-graduate training)<br><br>Doctoral degree (such as PhD., MD, EdD, DVM, DDS, or JD)<br><br>Prefer not to say<br><br>Don't know
**Household**	
How many **adults** (18 years and older **including yourself**) are permanent residents of your primary household? These terms refer to the home that you or other adults consider to be the place or address where they live.	__ [Enter number in text field]
How many **children** (less than 18 years) are permanent residents of your primary household **and** live there **more than half** of a typical month?	__ [Enter number in text field]
If other than "0": *Are any of these children* ≤ ***5 years old***?	Yes<br>No<br>Prefer not to say<br>Don't know
If YES: How many are ≤ ***5 years old?***	__[Enter number in text field]
If YES: How many are ≤ ***36 months old?***	__[Enter number in text field]
If YES: How many wear diapers or need help toileting most of the time?	_ [Enter number in text field]
If YES ≤ 36 months old: *How many of them attend a large group education or care program other than kindergarten?*	__[Enter number in text field]
If YES ≤ 36 months old: *How often do you play with them?*	Always<br>Often<br>Sometimes<br>Rarely<br>Never
If YES ≤ 36 months old: *How often do you hold them?*	Always<br>Often

	Sometimes<br>Rarely<br>Never
If YES ≤ 36 months old: How often do you kiss them near the mouth or nose?	Always<br>Often<br>Sometimes<br>Rarely<br>Never
If YES ≤ 36 months old: How often do you feed them?	Always<br>Often<br>Sometimes<br>Rarely<br>Never
If any except "never": How often do you share utensils, dishes, food, drinks, or straws with them?	Always<br>Often<br>Sometimes<br>Rarely<br>Never
If YES ≤ 36 months old: How often do you put a pacifier or toothbrush they've used into your mouth?	Always<br>Often<br>Sometimes<br>Rarely<br>Never
If YES ≤ 36 months old: How often do you change diapers or help them with toileting?	Always<br>Often<br>Sometimes<br>Rarely<br>Never
If YES ≤ 36 months old: How often do you change clothes possibly soiled with their saliva or urine?	Always<br>Often<br>Sometimes<br>Rarely<br>Never
If YES ≤ 36 months old: How often do you clean or bathe them?	Always<br>Often<br>Sometimes<br>Rarely<br>Never
If YES ≤ 36 months old: How often do you check temperatures or provide other care for them when they're sick at home?	Always<br>Often<br>Sometimes<br>Rarely<br>Never
If YES ≤ 36 months old: How often do you disinfect rooms, feeding areas, diapering/toileting areas, equipment, toys, surfaces, or other things that might have contacted their saliva or urine?	Always<br>Often<br>Sometimes<br>Rarely<br>Never

If <enter activity>, how often do you wear gloves while <enter activity>?	Always<br>Often<br>Sometimes<br>Rarely<br>Never
If <enter activity>, how often do you wash your hands after <enter activity>?	Always<br>Often<br>Sometimes<br>Rarely<br>Never
What languages are spoken by most members of your primary household?	English<br>Spanish<br>Other Please specify
**In the past year**, has a financially responsible adult(s) been almost or completely unable to pay for your primary household expenses? Include yourself if applicable.	Yes<br>No<br>Prefer not to say<br>Don't know
**In the past year**, has your primary household had difficulty accessing any of the following? Check all that apply.	Food<br>Heat<br>Running water<br>Electricity<br>Clothing<br>Medical care<br>Medication<br>Other<br>Prefer not to say<br>Don't know
Does anyone in your primary household have income-based government assistance of any kind?	Yes<br>No<br>Prefer not to say<br>Don't know
What is your primary form of health insurance?	Insurance through a current or former employer or union (yours or another family member's). This includes COBRA.<br><br>Insurance purchased directly from an insurance company (by you or another family member). This includes coverage purchased through an exchange or marketplace, such as HealthCare.gov.<br><br>Any kind of state or government-sponsored assistance plan based on income or disability<br>IF YES à

	Medicare for people 65 and older or people with certain disabilities.<br>Medicaid<br>Other Please specify<br><br>TRICARE or other military health care, including VA health care.<br><br>Indian Health Service.<br><br>No insurance coverage<br><br>Prefer not to say<br>Don't know
Are there any cultural, religious, or other practices in your household(s) that may involve contact with the child's saliva or urine?	Yes, Please describe<br>No<br>Prefer not to say<br>Don't know
**Workplace**	
When did you start working at this EEC center? If you're not sure, enter an approximate date.	[Enter date]
How many hours do you work at the center during an average week?	[Enter number in text field]
How many weeks per year do you work at the center?	52-39 weeks/year<br>38-27 weeks/year<br>26-13 weeks/year<br>5-14 weeks/year<br><5 weeks/year
Which statement(s) best describes your role at the center? Select all that apply.	Transporting children to or from center<br><br>Cooking or delivering meals for children or staff at center<br><br>Teaching or caring for children in the classroom<br><br>Feeding children meals or snacks<br><br>Cleaning or bathing children<br><br>Caring for sick children at the center<br><br>Management of center staff and/or operations<br><br>Administrative duties at the center<br><br>Maintenance or cleaning of the center facilities

	Cleaning toys, furniture, linens, or other items that children contact<br><br>Early intervention or other specialist services for children (such as PT/OT or music)
How many hours per week do you have **direct contact** with children ≤ **36 months old** while working **at this center** (such as changing diapers, feeding, holding, playing or other hands-on activities)?	__[Enter number in text field]<br>Not applicable
In which classroom(s) do you spend **most** (more than half) of your time at work?	[Text]
Select which activities you perform **most** (more than half) workdays. Select all that apply.	Holding children<br><br>Playing with children<br><br>Feeding children<br><br>Changing diapers or helping children with toileting<br><br>Changing clothes possibly soiled with children's saliva or urine<br><br>Cleaning or bathing children<br><br>Checking temperatures or other care for sick children at the center<br><br>Disinfecting rooms, feeding areas, diapering/toileting areas, equipment, toys, surfaces, or other things that might have contacted children's saliva or urine<br><br>None of the above<br><br>Other, Please specify
***If <enter activity>**, how often do you wear gloves while <enter activity>?*	Always<br>Very often<br>Sometimes<br>Rarely<br>Never
***If <enter activity>**, how often do you wash your hands after <enter activity>?*	Always<br>Very often<br>Sometimes<br>Rarely<br>Never

Does your center have a document, website, or other written resource outlining policies and procedures for infection control?	Yes<br>No<br>Prefer not to say<br>Don't know
How does your center train staff members about infection control? Check all that apply.	Live presentation (in person or remote)<br><br>Self-directed with recorded presentation<br><br>Self-directed with written information<br><br>Live presentation and self-directed<br><br>Center does not specifically train about infection control<br><br>Other, Please specify<br><br>Prefer not to say<br><br>Don't know
**Medical**	
**In the past 14 days**, have you had any of these symptoms? Check all that apply	Cough, runny nose, sore throat, trouble breathing, wheezing or other respiratory<br>Vomiting, diarrhea, belly pain, or other intestinal<br>Headache, joint, or other pain<br>Rash or other skin<br>Fever or other signs of infection<br>General symptoms such as fatigue, low appetite, muscle aches, or irritability<br>Other<br>None<br>Prefer not to say<br>Don't know
Have you **ever** been diagnosed by a healthcare provider to have wheezing, asthma, or any other lung or breathing condition?	Yes<br>No<br>Prefer not to say<br>Don't know
IF YES, have you been diagnosed **in the past 14 days?**	Yes<br>No<br>Prefer not to say<br>Don't know
Have you **ever** been hospitalized?	Yes<br>No<br>Prefer not to say

	Don't know
***IF YES,* In the past 6 months**, how many times were you hospitalized?	__ [Enter number in text field]<br>Prefer not to say<br>Don't know
IF YES, for what type of problem(s) were you hospitalized? Check all that apply	Cough, runny nose, sore throat, sinuses, ear pain, or other symptoms of a cold<br>Trouble breathing, wheezing or other symptoms in the chest<br>Vomiting, diarrhea, stomach pain, or other belly symptoms<br>Headache, joint, or other pain<br>Rash or other skin<br>Fever or other signs of infection<br>General symptoms such as fatigue, low appetite, muscle aches, or irritability<br>Other<br>Prefer not to say<br>Don't know
Have you ever been diagnosed by a healthcare provider to have a low immune system?	Yes<br>No<br>Prefer not to say<br>Don't know
Have you ever been tested for CMV?	Yes<br>No<br>Prefer not to say<br>Don't know
IF YES, when was the test done?	In the past 14 days<br>In the past 6 months<br>In the past year<br>More than 1 year ago<br>Prefer not to say<br>Don't know
IF YES, what was the result?	Positive<br>Negative<br>Inconclusive<br>Prefer not to say<br>Don't know
Have any of your household members ever been tested for CMV?	Yes<br>No<br>Prefer not to say<br>Don't know

IF YES, when was the test done?	In the past 14 days<br>In the past 6 months<br>In the past year<br>More than 1 year ago<br>Prefer not to say<br>Don't know
IF YES, what was the result?	Positive<br>Negative<br>Inconclusive<br>Prefer not to say<br>Don't know

Appendix 9. Family feedback survey.

Family Feedback Survey
REDCap Platform

**Instructions**	
We invite you to share your perspectives about the CMV TransmIT Study or research in general—***whether or not your child enrolled in this study***.<br><br>After starting this survey, you can choose to provide feedback in one or both of these ways:<br>1. Complete the survey. The survey could take up to 10 minutes and your responses are anonymous.<br>2. Attend a focus group. Focus groups are interactive discussions with other families. The sessions are about 60-90 minutes, but you can leave at any time.<br><br>However you prefer to provide feedback, we hope you feel comfortable sharing as many details as possible. Your feedback will directly impact the way we perform this research going forward.<br><br>Thank you for sharing your perspective with us!	
**Participation in the study**	
Did you participate in the CMV TransmIT study?	YES<br>NO
**Feedback Format (Choosing both will branch to survey then focus groups)**	
Please select your preferred option(s) for providing feedback.	I would like to complete the feedback survey only. [conditional branching logic based on first question]<br>I would like to attend a focus group only. [conditional branching logic based on first question]<br>I would like to complete the feedback survey and attend the focus group. [conditional branching logic based on first question]
***Branching logic:* YES (Survey only AND participated in study)**	
What were your main reasons for enrolling in the CMV TransmIT Study? Select all that apply and add any comments in "Other". We will use survey responses to improve the study based on feedback from families.	To help others<br>To advance research<br>To benefit my child<br>I know someone who has been sick from CMV<br>The gift card given for participation<br>I've heard about CMV from my healthcare provider<br>I've heard about CMV from the media (such as news, websites, or social media)<br>I've heard about CMV from my friends or family<br>I wanted to know more about CMV<br>I prefer not to say.<br>Other: [branching logic to fillable field]
Participating in the study was easy.	Strongly Agree<br>Agree<br>Somewhat Agree<br>Disagree

Family Feedback Survey V2 STUDY00000521 02OCT2024

Note: Only the first page of this document is included here for illustrative purposes. The complete version is available upon request.

Appendix 10. Participant and sample identification codes.

Stage 1

Child

Data fields

Participant ID:	CMV1-64828-84923
Sample type:	S, U, B
Time point:	V#

Sample ID
64828-84923.(S,U,B).V#

→

Stage 2

Child

Data fields

Participant ID:	CMV2-12423-25442
Stage 1 ID:	CMV1-64828-84923
Household ID:	GTHSW
Participant type:	C
Sample type:	S, U, B
Time point:	V#

Sample ID
12423-25442.GTHSW.C.(S,U,B).V#

Primary Caregiver

Data fields

Participant ID:	CMV2-76231-94648
Household ID:	GTHSW
Participant type:	PC
Household member:	1
Sample type:	S, U, B
Time point:	V#

Sample ID
76231-94648.GTHSW. PC.(S,U,B).V#

Household Member

Data fields

Participant ID:	CMV2-64746-29457
Household ID:	GTHSW
Participant type:	HM
Household member:	≥2
Sample type:	S, U, B
Time point:	V#

Sample ID
76231-94648.GTHSW.HM1.(S,U,B).V#

B, blood; C, child; CMV, cytomegalovirus; EEC, early education and care; HM, household member; ID, identifier; GTSHW, non-descriptive household ID; PC, primary caregiver; S, saliva; U, urine; V#, visit number.

Appendix 11. Selection process and criteria for collection of saliva samples.

(**A**) Criteria were developed through consultation with the Kids Comfort Promise team from the University of Massachusetts Memorial Health Children's Medical Center to select the most tolerable device for sample collection from children.

Selection criteria for sample collection devices
• Safe for children up to 36 months of age, especially in case of biting and for children with oral or facial abnormalities (e.g. uncorrected cleft lip or palate)
• Approved for use in children
• Minimally distressing for participants
• Parents or participants with no prior knowledge, limited education level, and intellectual or physical disability, or other potential challenges able to understand the instructions and operate the device
• High saliva volume capacity of absorbent pad
• Cells and proteins minimally filtered out
• Acceptable ratio of sample to viral transport medium to sustain viable virus
• Easy for parents and center staff to collect the sample at home, particularly in difficult settings (e.g. noise, limited space, or other children nearby)
• Reasonable cost
• Feasible to package into a home sampling kit

(**B**) Saliva collection devices (Oasis) selected and used in the study for collecting samples from children (left) and adult early education and care center staff (right).

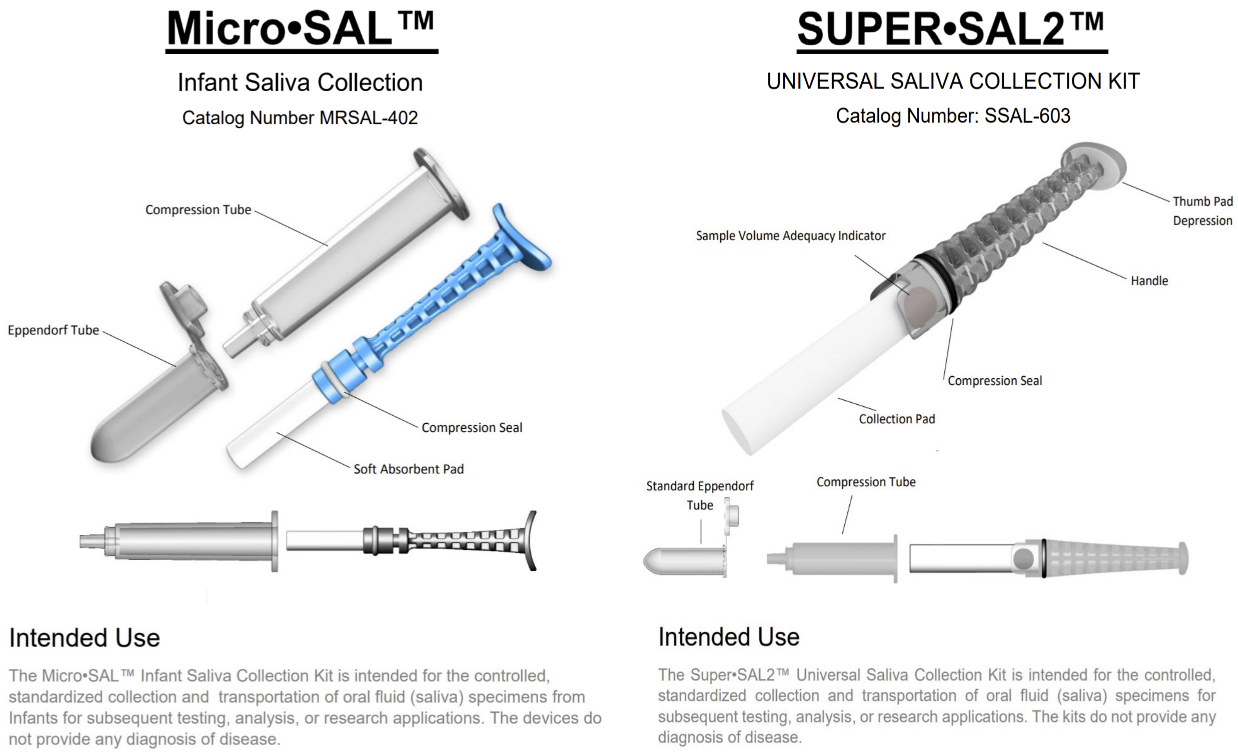

Appendix 12. Home sample collection instructions for the pilot study. (**A**) Children. (**B**) EEC center staff

(A)

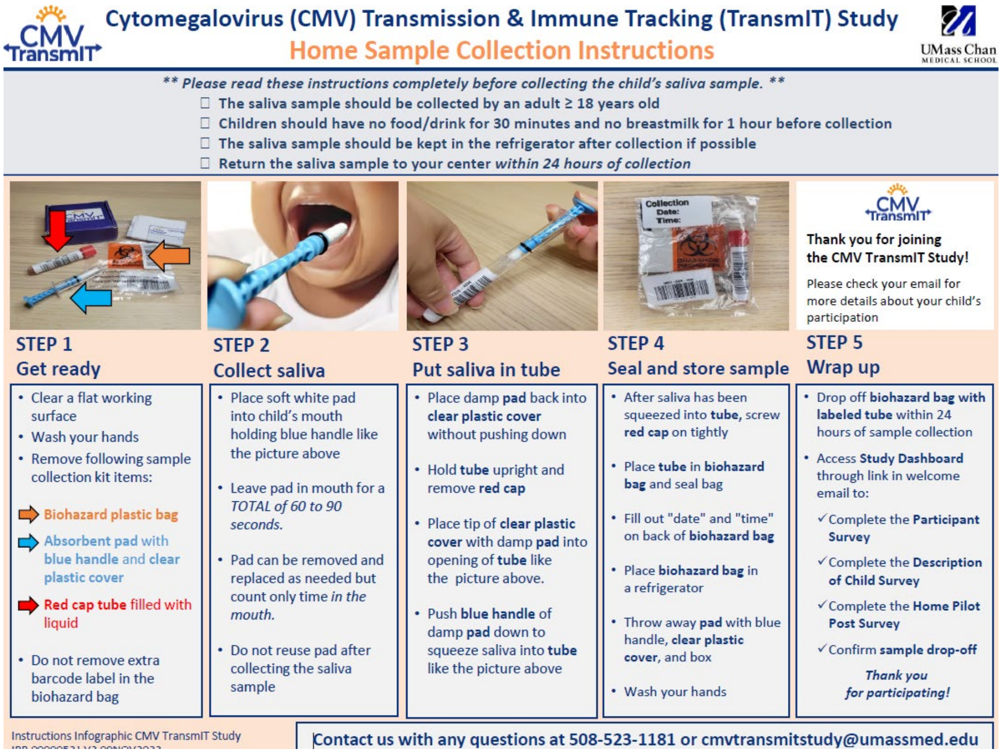

(B)

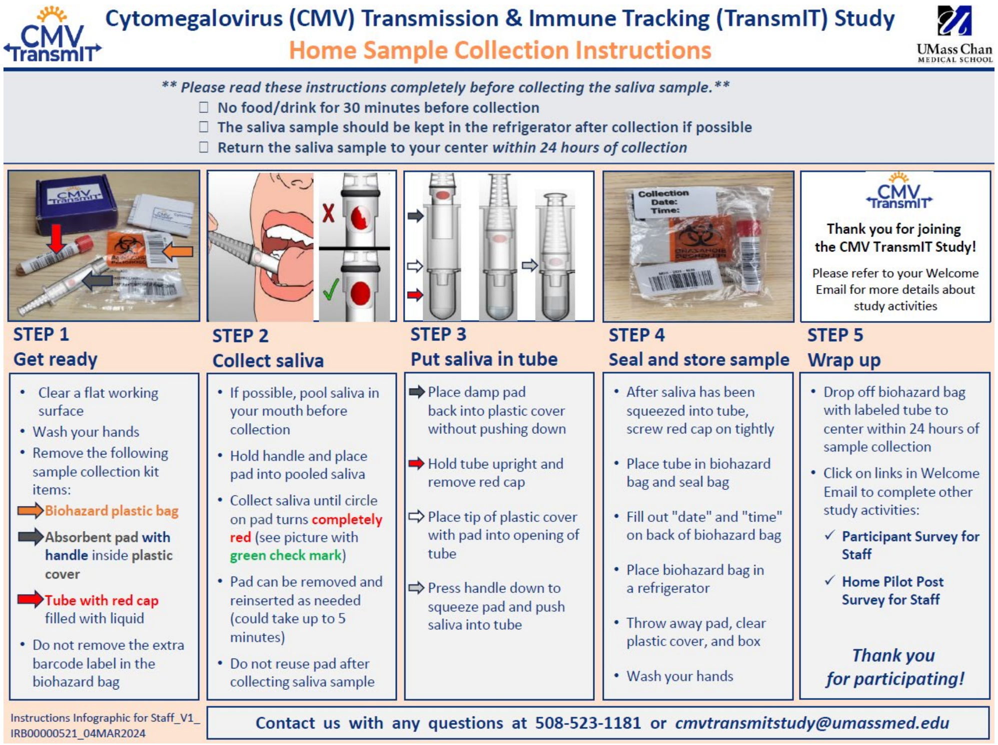

Appendix 13. Experience of the home sample collection. (**A**) Description of the child during sample collection. **B**) Post-pilot survey for children. (**C**) Post-pilot survey for EEC center staff member

(A)

CMV TransmIT Study

Description of the Child During Sample Collection

Please complete these questions about the child at the time of saliva collection.

1. Getting ready for sample collection

   a. Has the child had anything to eat or drink < 30 minutes before saliva collection?
      - Yes
      - No
      - Prefer not to say
      - Don't know

   b. Does the child have any medical condition or disability that might affect the saliva collection process?
      - Yes
        - If yes → Please describe
      - No
      - Prefer not to say
      - Don't know

   c. Do you notice any of these signs? Check all that apply
      - cuts/bleeding, redness/swelling, or spots (such as sores, bumps, pimples) on the lips or skin around the mouth
      - **cuts/bleeding, redness/swelling, white coating, or spots (such as sores,** bumps, pimples) of the gums, tongue, or inner cheeks
      - dryness inside the mouth
      - Prefer not to say
      - Don't know
      - If any boxes checked → Please take a picture, upload to MyDataHelps, and add any comments or contact study staff at (508) 523-1181

2. During and after sample collection

   a. Did you notice any **new** blood or other signs of injury in or around the mouth?
      - Yes
      - No
      - Prefer not to say
      - Don't know

Home Pilot Description of the Child Form IRB 00000521 CMV TransmIT_V3_11JUN2024

Note: Only the first page of this document is included here for illustrative purposes. The complete version is available upon request

(B)

CMV TransmIT

UMass Chan MEDICAL SCHOOL

Thank you for participating in our pilot study for home sample collection!

To make sure we offer options that work for families, we would like to learn more about your experience with sample collection at home.

Please take 3-5 minutes to complete this Home Pilot Post Survey. If possible, the adult(s) who collected the sample from the child, completed the Description of Child Survey in the Study Dashboard and/or dropped the sample at the center should fill it out.

1. What was your **overall experience** collecting the saliva sample at home? Take into account 1) picking up the Sample Collection Kit, 2) collecting the sample from the child, 3) completing the **Description of Child Survey in the Study Dashboard**, and 4) returning the sample to the center.

   Positive
   Neutral
   Negative

   [Branching logic for positive]
   What contributed to your positive overall experience of the home collection process? Check all that apply and/or select "Other" to add any comments.

   Clear guidance from study staff and/or written instructions
   Sample Collection Kit items easy to handle
   Took only a short time
   Easy to perform
   Convenient
   Few distractions at home
   Child was in a more comfortable place and around familiar people (compared to the center)
   Other – Please comment

   [Branching logic for negative]
   What contributed to your negative overall experience of the home collection process? Check all that apply and/or select "Other" to add any comments.

   Unclear guidance from study staff and/or written instructions
   Sample Collection Kit items difficult to handle
   Took too much time
   Difficult to perform
   Inconvenient
   Many distractions at home
   Child was in a less comfortable place and around unfamiliar people (compared to the center)
   Other - Please comment

2. How would you rate the difficulty of **understanding the instructions** for saliva collection at home?

Home Pilot Post Survey CMV TransmIT Study IRB 00000521 V2.0 30NOV2023

Note: Only the first page of this document is included here for illustrative purposes. The complete version is available upon request.

(C)

Thank you for participating in our pilot study for home sample collection!

To make sure we offer options that work for center staff who enroll in our study, we would like to learn more about your experience with sample collection at home.

Please take 3-5 minutes to complete this Home Pilot Post Survey.

1. Did you have anything to eat or drink 30 minutes before saliva collection?
   Yes
   No

2. Do you have any medical condition or disability that might have affected the saliva collection process?
   Yes
   No
   Prefer not to say
   Don't know

3. Did you notice any cuts, bleeding, sores, bumps, rashes, or other signs of injury or infection in or around your mouth ***before*** collection?
   Yes, Please describe
   No
   Prefer not to say
   Don't know

4. Did you notice any **new** blood or other ***new*** signs of injury in or around the mouth ***after*** collection?
   Yes, please describe.
   No

   [Branching logic if yes]
   Please contact study staff at 774-772-4905

5. What was your **overall experience** collecting the saliva sample at home? Take into account 1) picking up the Sample Collection Kit, 2) collecting the sample, and 3) returning the sample to the center.

   Positive
   Neutral
   Negative

   [Branching logic for positive]
   What contributed to your positive overall experience of the home collection process? Check all that apply and/or select "Other" to add any comments.

   Clear guidance from study staff and/or written instructions

Home Pilot Post Survey for Staff_V1_IRB00000521_04MAR2024

Note: Only the first page of this document is included here for illustrative purposes. The complete version is available upon request.
